# Molecular Dynamics Simulations of the Bacterial UraA H^+^-Uracil Symporter in Lipid Bilayers Reveal a Closed State and a Selective Interaction with Cardiolipin

**DOI:** 10.1371/journal.pcbi.1004123

**Published:** 2015-03-02

**Authors:** Antreas C. Kalli, Mark S. P. Sansom, Reinhart A. F. Reithmeier

**Affiliations:** 1 Department of Biochemistry, University of Oxford, Oxford, United Kingdom; 2 Department of Biochemistry, University of Toronto, Toronto, Canada; Max Planck Institute for Biophysical Chemistry, Germany

## Abstract

The *Escherichia coli* UraA H^+^-uracil symporter is a member of the nucleobase/ascorbate transporter (NAT) family of proteins, and is responsible for the proton-driven uptake of uracil. Multiscale molecular dynamics simulations of the UraA symporter in phospholipid bilayers consisting of: 1) 1-palmitoyl 2-oleoyl-phosphatidylcholine (POPC); 2) 1-palmitoyl 2-oleoyl-phosphatidylethanolamine (POPE); and 3) a mixture of 75% POPE, 20% 1-palmitoyl 2-oleoyl-phosphatidylglycerol (POPG); and 5% 1-palmitoyl 2-oleoyl-diphosphatidylglycerol/cardiolipin (CL) to mimic the lipid composition of the bacterial inner membrane, were performed using the MARTINI coarse-grained force field to self-assemble lipids around the crystal structure of this membrane transport protein, followed by atomistic simulations. The overall fold of the protein in lipid bilayers remained similar to the crystal structure in detergent on the timescale of our simulations. Simulations were performed in the absence of uracil, and resulted in a closed state of the transporter, due to relative movement of the gate and core domains. Anionic lipids, including POPG and especially CL, were found to associate with UraA, involving interactions between specific basic residues in loop regions and phosphate oxygens of the CL head group. In particular, three CL binding sites were identified on UraA: two in the inner leaflet and a single site in the outer leaflet. Mutation of basic residues in the binding sites resulted in the loss of CL binding in the simulations. CL may play a role as a “proton trap” that channels protons to and from this transporter within CL-enriched areas of the inner bacterial membrane.

## Author Summary

Symporters are proteins that are responsible for the co-transport of ions and small molecule solutes across cell membranes. UraA is an example of a symporter, and is responsible for the proton-driven uptake of uracil in bacteria like *E*. *coli*. Despite its importance as a member of a large family of nucleobase/ascorbate transporters (NAT) and the existence of structural and functional data, the mechanism by which UraA transports uracil across the bacterial membrane, and in particular the role of its diverse and complex lipid environment in the transport mechanism, remains elusive. In this study, we have used a multiscale computational methodology to examine the dynamics of UraA and to elucidate its interactions with lipids that resemble its native environment in the bacterial inner membrane. Our results demonstrate that negatively-charged lipids in the membrane (phosphatidylglycerol and cardiolipin) associate preferentially with UraA and may play a role in its function. Additionally, our simulations resulted in a closed state of UraA, a likely intermediate in the transport mechanism that may not be readily accessible by experimental methods.

## Introduction

Crystal structures of membrane proteins, particularly from bacterial sources, are being determined at an increasing rate (http://blanco.biomol.uci.edu/mpstruc/). The membrane protein is typically isolated and crystallized in the presence of detergents and indeed, non-native detergent molecules are often found tightly associated with the membrane protein. It is therefore important to relate the crystal structure back to the state of the protein in a native lipid bilayer [1,2].

Molecular dynamics (MD) simulations of membrane proteins in increasingly complex lipid bilayers [3] can now be applied with confidence to build dynamic models of membrane proteins in a native milieu [4,5]. A key question to be explored is to what extent the crystal structure determined in the presence of detergents is the same as the structure of the membrane protein in a lipid bilayer. For example, the influenza M2 protein transmembrane domain shows significant structural differences between detergent-containing crystals and the structure in lipid bilayers determined by solid state NMR [1]. Furthermore, there are a number of examples of membrane protein structures that contain tightly associated lipids bound to specific sites on the protein [6,7]. MD simulations allow us to explore molecular motions and the dynamic interactions between lipids and proteins, providing a complementary approach to the temporal and spatially averaged static structures determined by X-ray crystallography. Recent studies using MD simulations have identified cardiolipin (CL) binding sites in the cytochrome bc_1_ transporter [8] and in the cytochrome c oxidase [9], and PIP_2_ binding sites in Kir channels [10,11]. MD simulations were also used to identify interactions of lipids with the aquaporin family [12,13] and other proteins [14,15]. Recently MD simulations were also used to study the conformation of proton-driven transporters LacY [16] and UpaA [17]. So, how close is the structure of a membrane protein in a lipid bilayer determined by MD simulations to the original structure of the protein determined by X-ray crystallography in the presence of detergents? Can we use MD simulations to detect specific lipid-protein interactions not often found in crystal structures and study their dynamics?

**Fig. 1:**
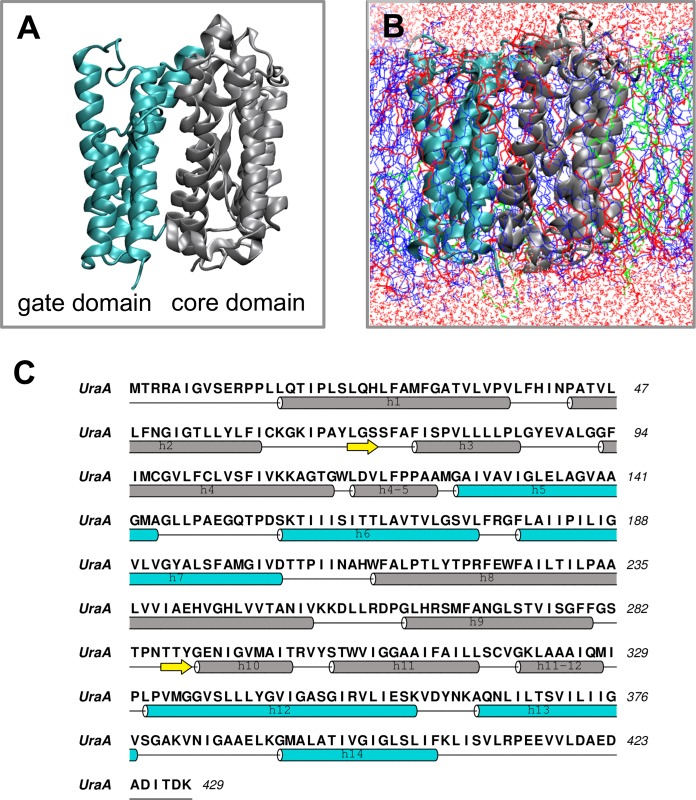
Structure of UraA. A. The core and the gate domains as suggested by *Lu et al*. [20] are shown in grey and cyan respectively. B. Snapshot from the end of one of the UraA-AT simulations. The same colors for UraA as in A are used. The POPE, POPG and CL are shown in blue, red and green respectively. The water solvent is shown in red. C. Sequence of UraA. The secondary structure is shown below the sequence. The same colors for the core and the gate domains as in A are used.

We explored these questions using the *E*. *coli* UraA H^+^-uracil symporter, the crystal structure of which was determined with bound uracil in the detergent n-nonyl-β-D-glucopyranoside (NG; Fig. 1). UraA is a member of the nucleobase/ascorbate transporter NAT family of proteins (also known as nucleobase-cation symporter-2 [NCS2]) which can be found in all species [18]. The majority of the NAT proteins are responsible for the uptake of xanthine, uric acid or uracil with H^+^ in the case of most bacteria and Na^+^ in the case of the mammalian transporters [17,18]. In mammals, NAT proteins are also responsible for the transport of vitamin C [18]. Including UraA, there are ten members of the NCS2 family of transporters in *E*. *coli*. Extensive mutagenesis and *in silico* studies identified a conserved 11-residue NAT motif in the NAT/NCS2 family ([Q/E/P-N-x-G-x-x-x-x-[RKG]) that is crucial for the function of its members. Mutation of the conserved residues of the motif in the *Aspergillus nidulans* uric acid/xanthine permease, UapA and the *E*. *coli* xanthine permease XanQ (YgfO) have shown that residues within the NAT motif contribute to the substrate specificity [19]. Extensive cysteine-scanning mutagenesis studies on XanQ have identified key functional residues, including Gln324 and Asn325 in the NAT motif within TM10 [19].

The 2.8 Å crystal structure of the UraA uracil transporter (Fig. 1), the first member of the NCS2 family to be solved, revealed a monomer protein with 14 transmembrane segments with TM segments 1–7 and TMs 8–14 having an inverted topology [20]. Uracil was located in a central cavity open via a water-filled passage to the cytosolic side of the membrane, bound between a pair of short anti-parallel β-strands in TM3 and 10 via a predominant interaction with an invariant Glu241 [20]. The crystal structure was obtained in the detergent n-nonyl-β-d-glucopyranoside with one inverted detergent molecule associated with the protein with the glucose head group of the detergent donating a hydrogen bond to the carbonyl in uracil, thereby stabilizing its binding.

In this study a serial multiscale MD simulation approach [21] was used to study the dynamics of the UraA (3QE7), simulated without bound uracil or detergent in a native-like lipid bilayer, enabling us to explore its specific interactions with the lipids. To this end, a lipid bilayer containing POPC lipids was self-assembled around UraA and then simulations in pure POPC, pure POPE and a POPE (75%), POPG (20%) and CL (5%) mixture to mimic the native bacteria membrane in the coarse-grained and atomistic resolutions were performed (see Methods, Table 1 and S1 Table for more information). Simulations in the absence of substrate (and bound detergent) resulted in a closed state of the protein. The various lipid bilayers that we have used allowed us to study the differences in the conformational states of UraA in different lipid environments. We discovered a preferential interaction of anionic lipids PG and CL with UraA. In particular, two CL sites were identified on UraA on the inner leaflet and a single CL site in the outer leaflet. The inner association involved a preferential interaction of the phosphate oxygen of the CL head group with R299 and with R4 near the cytosolic exit site of the transporter at one site, and with R265 and K109 at the second site. CL also showed a preferential interaction with UraA on the outer leaflet involving K321 and W212 in the third site. *In silico* mutation of these basic residues led to loss of CL binding highlighting the importance of an electrostatic interaction in the association of this anionic lipid with UraA.

**Table 1:** Summary of the principal simulations.

Simulation	Description	Bilayer Concentration	Duration
UraA-CG	UraA (PDB: 3QE7)	POPE(75%)/POPG(20%)/CL(5%)	7 x 1 μs, 3 x 10 μs
UraAmut-1-CG	UraA [mutations: R4A, R299A, K321A]	POPE(75%)/POPG(20%)/CL(5%)	10 x 1 μs
UraAmut-2-CG	UraA [mutations: K157A, R222A, K321A, K381A, K390A]	POPE(75%)/POPG(20%)/CL(5%)	10 x 1 μs
UraAmut-3-CG	UraA [mutations: R4A, R11A, K62A, K109A, K110A, R177A, R265A, R299A, R351A, K357A, K362A, K407A]	POPE(75%)/POPG(20%)/CL(5%)	10 x 1 μs
UraA-AT	UraA	POPE(75%)/POPG(20%)/CL(5%)	2 x 0.1 μs 2 x 0.5 μs

This table provides the principal simulations; further details of all other simulations are provided in S1 Table. All the simulation systems are described in detail in Methods. The simulation protocol is also shown in S1 Fig.

## Methods

### Coarse-grained molecular dynamics simulations

The simulation protocols used in the study are summarized schematically in S1 Fig. Coarse-grained molecular dynamics (CG-MD) simulations were performed using the MARTINI force field [22]. In the MARTINI CG model used [22,23] four heavy atoms (i.e. non-hydrogen) are represented as one CG particle. Four CG particle types exist: charged (Q), non-polar (N), apolar (I) and polar (P). Additional particle subtypes exist for assigning H-bond capabilities. Each amino acid is represented by the aforementioned particles representing the alpha carbon and the various portions of the side chain [22,23]. An elastic network was applied to all backbone particles using a cut-off distance of 7 Å to model secondary and tertiary structure. The use of an elastic network model (ENM) to maintain protein tertiary structure during the CG-MD simulations restricts any major conformational changes of the protein. However, during the subsequent atomistic simulations the ENM is removed. In the CG-MD simulations the original MARTINI water model [22,23] was used, without addition of antifreeze particles to any of the systems.

The starting structure of the UraA transporter, without the bound uracil or single detergent molecule, was taken from the protein coordinates of 2.8 Å crystal structure deposited in the PDB (3QE7). The CG protein was centered in a 14 x 14 x 12 nm^3^ simulation box of sufficient size to accommodate the 429-residue protein monomer (45 kDa) and about 400 phospholipid molecules. UraA was oriented to align the transport path of the protein with the z axis of the simulation box with the bottom of the structure facing the cytosol and the top the periplamic space. UraA contains 14 transmembrane segments and both the N- and C-termini face the cytosol.

**Insertion of UraA in a lipid bilayer.** POPC lipids were placed randomly in the simulation box and the system was solvated with CG water molecules and NaCl ions to ∼150 mM were added to neutralize the system. In particular 296 Na^+^ and 176 Cl^-^ ions were added to neutralize the -84*e* charge of the POPG lipids, the -42*e* charge of the CL and the +6*e* net charge of the protein. These ions were used to neutralize the systems, and no specific interactions between the ions and the lipids or the protein were observed. The system was energy-minimized using the steepest descent algorithm embedded in GROMACS. Subsequently, a CG-MD simulation for 100 ns was run which allowed the lipids to self-assemble forming a bilayer around the membrane protein (S1 Video). Note that during the self-assembly simulation the protein Cα atoms were restrained so the protein backbone could not move, while the side chains were mobile. At the end of the 100 ns self-assembly CG-MD simulation with UraA now in a bilayer, the protein restraints were removed and further 1 μs of production simulation with the protein free to diffuse in the bilayer was run. This allowed the protein to optimize both its position in the lipid bilayer (e.g. optimize the protein displacement and tilt angle relative to the bilayer) and its interaction with surrounding lipids.

**Lipid exchange.** At the end of the unrestrained 1 μs simulation in the POPC bilayer the protein was re-centered in the simulation box and the POPC lipids were exchanged for 100% POPE, or 75% POPE, 20% POPG, 5% CL [24] using a locally written script (see Table 1 and S1 Table). The script randomly replaces one POPC molecule (in the case of POPE or POPG) or two POPC molecules (in the case of CL, which consists of two diacylphosphatidic acid moieties connected by a central glycerol) with the desired number of POPE and/or CL molecules in each leaflet thus creating a new bilayer where the lipids are randomly distributed in the two leaflets in the desired concentrations. After lipid exchange the systems were energy minimized followed by 5 ns of equilibration at 323K with the protein Cα atoms restrained. It should be emphasized that in the latter bilayer the lipids were randomly distributed between the two leaflets. Seven independent repeat production simulations for 1 μs each and three independent repeat production simulations for 10 μs each were performed starting with different initial configurations (i.e. with the same initial position but with different initial velocities) to study the dynamic interactions of UraA with native lipids. For the pure POPE bilayer only 1 simulation was performed (for 1 μs).

**Mutant forms of UraA.** Simulations using the aforementioned system with UraA in the 75% POPE, 20% POPG, 5% CL bilayer were also performed using mutated forms of the protein to study the role of specific residues in binding lipids (see Table 1 for more information). Additionally, removing basic residues from the CL binding sites of UraA facing the outer bilayer leaflet (UraAmut-2-CG), facing the inner bilayer leaflet (UraAmut-3-CG) or a combination of the two (UraAmut-1-CG) allowed us to probe if CL molecules bound cooperatively to the three CL binding sites. All mutations were performed using Modeller [25,26]. In particular, in UraAmut-1-CG residues R4 and R299 in site 1 and K321 in site 3 were mutated to alanines. In UraAmut-2-CG residues K157, R222, K321, K381, and K390 in or close to site 1 were mutated to alanines. These mutations removed the CL binding site in the outer leaflet of the bilayer. In UraAmut-3-CG residues R4, R11, R177, R299, R351, R357 and R362 in or close to site 1 and K62, K109, K110, R265 and K407 in or close to site 2 were mutated to alanines. These mutations removed the CL binding sites in the region of UraA facing the inner leaflet of the bilayer. Finally, simulations with mutations restricted to each of the three CL-binding sites: i) K321A, ii) K109A/R265A and iii) R4A/R299A were also performed. Note that in many cases more than one basic residue in the CL binding site was mutated to alanine in order to change the net positive charge of the CL binding site to neutral. This allowed us to prevent the CL molecules from interacting with other positive residues in the CL binding site, thus compensating for the loss of the main interacting residues. In these simulations, after the exchange of the lipids, the wild-type form of UraA was replaced by the mutated form and 10 individual repeat production simulations of 1 μs each were performed for each mutant.

**CG-MD simulations.** All CG-MD simulations were performed using GROMACS 4.5 (www.gromacs.org). A Berendsen thermostat (coupling constant of 1.0 ps; reference temperature 310 K for POPC and POPE lipid bilayer and 323 K for the more physiological lipid mixture) and barostat (coupling constant of 1.0 ps; reference pressure 1 bar) were used. The integration time step was 20 fs. Lennard–Jones and Coulombic interactions were shifted to zero between 9 and 12 Å, and 0 and 12 Å, respectively, following the original MARTINI parameterization.

### Atomistic molecular dynamics simulations

Coarse-grained (CG) to atomistic (AT) conversion was performed using the CG2AT protocol as described previously by Stansfeld *et al*. [27]. In this approach the lipids are reconstructed by aligning AT lipid fragments with the corresponding CG fragments from an energy-minimized library of atomistic lipid conformations. The protein backbone is reconstructed by the CG backbone trace using PULCHRA [28] and the side chains using Modeller [25,26]. Atomistic molecular dynamics (AT-MD) simulations were performed using the GROMOS96 53a6 force field that has been widely used in simulation studies of membrane proteins. The Parrinello–Rahman barostat [29] and the V-rescale thermostat [30] were used for pressure and temperature coupling, respectively. The LINCS algorithm [31] was used to constrain bond lengths, and the Particle Mesh Ewald (PME) algorithm [32] was used to model long-range electrostatic interactions. Lipid parameters for POPE and CL were from [33] and for POPG were from [34]. All atomistic systems were equilibrated for 2.5 ns with the protein backbone Cα atoms restrained, followed by unrestrained atomistic MD simulations from 100 to 500 ns with a time step of 2 fs. Note that the removal of uracil is necessitated by the lack of accurate simulation parameters for this ligand.

**Analyses**. All the analysis was performed using GROMACS [35,36], VMD [37] and locally-written scripts. Structures were visualized using VMD [37] and PyMol (http://www.pymol.org). Analysis of the lifetime of interactions between the different lipid types and the 3 CL binding sites was performed by calculating distances (d_ij_) between each binding site (*i*) and the head group of each of the 3 different lipid types (*j*). To define a contact a cut-off distance (δ) of 0.7 nm for POPE and POPG was used. For the CL lipids instead of using the standard 0.8 nm cutoff distance we have evaluated the optimal cutoff distance (δ) over a range of distances from 0.8 to 1.6 nm. This was done to reflect the larger headgroup of the CL molecules and additionally to take into consideration “rattling in a cage” motion of CL molecules. This motion of CL molecules has been reported in other CG-MD studies of these lipids [8]. Whilst the calculation of the lifetime of interactions is sensitive to the cutoff distance used, the trend that CL molecules form more prolonged interactions with CL binding site 1 is retained for all cutoff distances used. In particular, the longest lifetime of interactions of the CL molecules binding to CL site 1 for simulation 3 varies from ∼200 ns to ∼800 ns when varying δ. Similarly, for CL binding site 2 it varies from ∼200 ns to ∼450 ns, and for CL binding site 3 varies from ∼100 ns to ∼300 ns. A comparable sensitivity of the residence time has been observed to other studies which used CG-MD simulations to probe the CL interactions with membrane proteins [8]. Note that in all Figures/Tables we show the results obtained using a cutoff distance of 1.1 nm to define a contact for CL lipids. An occupancy variable ζ_ij_(t) was determined, defined as:

$$\begin{array}{l} \zeta_{ij}\left(t\right)=1\text{ if }d_{ij}\left(t\right)\leq \delta \;\text{or}\\ \zeta_{ij}\left(t\right)=0\;\text{if}\;d_{ij}\left(t\right)>\delta \end{array}$$

Thus if the distance between the lipid and the CL binding site was lower that the cut-off distance the occupancy was 1 otherwise the occupancy was 0 (see S2A Fig. for this analysis of one of the 10 μs simulations of UraA).

Using this measure of occupancy the stretches of time for which a site was continuously occupied by a specific lipid type were determined. For this analysis the trajectory was sampled every 0.3 ns, and we did not perform any smoothing of our raw data to remove any fast binding/unbinding events. This was because we concerned that smoothing of our trajectories would prevent us from capturing the faster binding/unbinding events of POPE and POPG lipids compared to CL, and additionally it has been suggested that application of different smoothing times of a trajectory may yield significantly different results [8]. For all lipid types we have observed some fast binding/unbinding events (shown in S2A Fig.), but for our analysis we have focused on the most extended continuous periods of interactions for each lipid (S3B Fig.). Finally, note that we present all of our results without any normalization in terms of the number of lipids in the bilayer. To define the CL binding sites we have used two residues in each site for which our contact analysis had revealed that were predominantly involved in the interactions with the lipids (site 1: R4 and R299; site 2: K109 and R265; site 3: K321 and W212; see also S2 Video). Note that using a two-residue representation of the CL binding site often allows more than one lipid to occupy the CL binding site. After we have extracted the periods of time for which a binding site was continuously occupied by a specific lipid type, we have used these data to calculate how many changes of single lipids of the same lipid type occurred in the CL binding site during the time of which the site was continuously occupied by a specific lipid type. This analysis is shown and discussed in detail for one of the simulations in S2 Fig. It should be noted, however, that the nature of the CG-MD simulations is such that calculated lifetimes of interactions should be viewed as indicative rather than absolute predictions.

In order to quantify the enrichment of anionic lipids (i.e. POPG and CL) around the protein we have merged the trajectories from the three extended CG-MD simulations, and have then calculated the mean density of each of the lipid types on the bilayer xy plane (S4 Fig.). The density was calculated for each leaflet separately. A region with a radius of 3 nm from the protein center of mass (representing the region adjacent to the protein) and a region with a radius of 3.5 to 6.0 nm from the protein center of mass (representing the bulk region of the bilayer) were selected (see S4 Fig.). The change in the density of each lipid type between the region adjacent to the protein and the bulk region of the bilayer was calculated by dividing the mean density of the lipids in the annulus close to the protein over the mean density of the lipids in the annulus representing the bulk region:

$$S=\frac{\mathrm{mean density}\;\left(0-3\;nm\right)}{\mathrm{mean density}\;(3.5-6\;nm)}$$

Therefore if the value of S is above 1 then it means that there is enrichment of the specific lipid type around the protein whereas if S is < 1 it suggests that the density of the lipids in the bulk region is higher compared to the density of the lipids close to the protein. Note that for the CL molecules, because of their preference for the CL binding sites, we have also calculated S using only the mean density of CL molecules in the CL binding sites.

## Results

### Cardiolipin (CL) binding sites on the UraA transporter

To study the preferential interaction of UraA with the anionic lipids in a membrane, the crystal structure of UraA (3QE7) was assembled in a lipid bilayer that resembles its native environment in the bacterial inner membrane. In particular, UraA in a lipid bilayer containing POPE (75%), POPG (20%) and CL (5%) was subjected to MD simulations at coarse-grained and atomistic resolutions (see Methods and Table 1 for more information). Note that in all simulations the uracil molecule was absent, as was the detergent molecule present in the crystal structure, producing an empty carrier. A key goal of this study was to explore the conformation of the empty carrier, which is an intermediate in the transport mechanism.

**Fig. 2:**
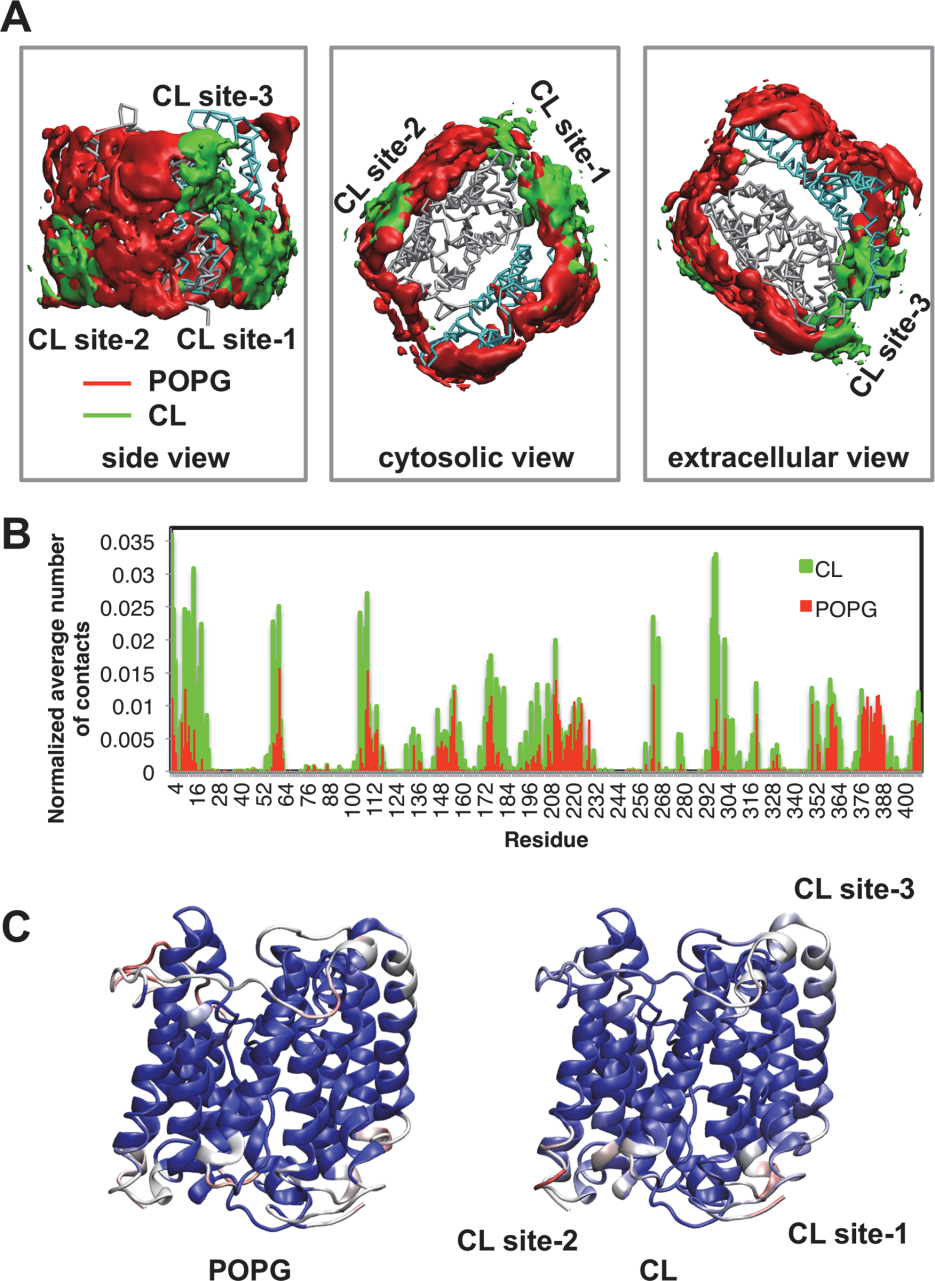
Interactions of UraA with lipids. A. Occupancy plots showing the probability of occurrence of cardiolipin (CL; green) and of the POPG (red) lipids around UraA. The occupancy was calculated as the average over all repeat coarse-grained simulations of the wild type UraA (UraA-CG in Table 1). Three different views are shown: a side view (left) showing the three CL binding sites, a cytosolic view facing the inner leaflet (middle) showing CL sites 1 and 2, and an extracellular view facing the outer leaflet of the bilayer (right) showing CL site 3. The distribution of all lipids separately is shown in S8 Fig. B. Normalized average number of contacts (using a cut-off distance of 7 Å for POPE and POPG and 8 Å for CL molecules) between the UraA and the head groups of POPG and CL lipids in the bilayer (across all repeats of the extended UraA-CG simulations; see Table 1). For the normalization, the number of contacts of a residue with a lipid type was divided by the number of lipids, the number of frames and the ratio of cutoff volumes. C. The number of contacts from one of the CG-MD simulations was mapped on the UraA crystal structure. Blue indicates a low number, white indicates a medium number and red a large number of contacts.

**Fig. 3:**
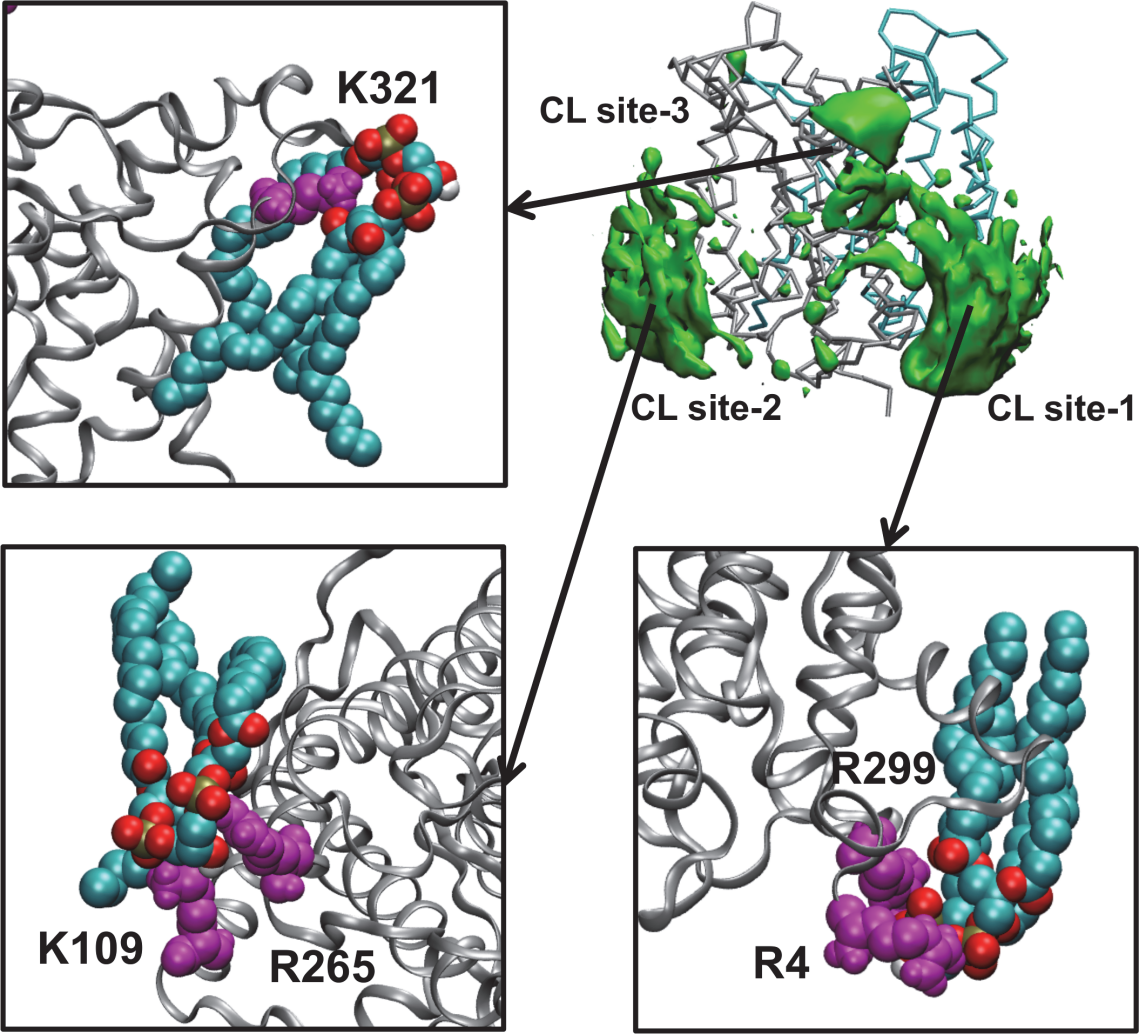
CL binding sites. Atomistic simulations of UraA in a 75% POPE/ 20% POPG/ 5% CL bilayer. Snapshots from the specific interactions between the CL and UraA are shown for the three CL binding sites (shown in green). The protein is represented as grey ribbons. The positively charged residues that form the main interactions with the CL in each CL binding site are shown in magenta. CL is shown in VDW format. Note that for this Figure we show snapshots with only CL lipids bound in the CL binding sites. As detailed in S4 Fig. and S6 Fig. and the in the text, POPG and POPE molecules to a lesser extent can also associate with the CL binding sites.

Analysis of the lipid densities around the protein during the CG-MD simulations revealed a high density of acidic lipids in the vicinity of the protein (Fig. 2). In particular, we have identified three main preferential sites in UraA that interact with the CL. In all CL binding sites lysine or arginine residues interact with the phosphate groups of the lipid head groups. These sites are shown in Fig. 2 and Fig. 3 and were reproducible in the repeat CG-MD simulations performed with the wild-type UraA. One of these sites (site 3) is located in the extracellular side of the protein at residue K321, which forms the main interactions with the phosphate group of CLs (formed mainly by TM helix 11, 12 and the unstructured region between helices 7 and 8). Other residues in this CL binding site that form significant interactions with the CL headgroup are V202 to T204 and I208 to W212 (Fig. 2 and Fig. 3). The remaining two CL binding sites (sites 1 and 2) are located in the cytosolic side of the protein, which unlike the UraA extracellular part, has many lysine and arginine residues in the lipid-exposed area of the protein creating a net positive surface (S5 Fig.). CL binding site 1 is located around residues R4, A5, I6, P13, F176, R177, G178 and A296 to V300 (TM helices 1, 7 and the loop region between helices 10 and 11 that contains R299). CL binding site 2 is located around residues C61, K62, I107 to A111 and R265 (formed by TM helices 2, 4 and 9). Other dynamic interactions of CLs with other parts of the protein were also observed (see S6 Fig. for detailed analysis of all the interactions). Calculation of the average number of CLs in contact with the protein during the simulations suggested that 2–3 CLs were in contact most of the time with the protein (S7 Fig.). Visualization of the final snapshots from all the individual repeat CG-MD simulations (Fig. 3) showed that in the vast majority of the simulations one CL lipid was associated with each CL binding site (see also S3 Video). To test if the interactions between the CL molecules and UraA are retained on longer timescales, three simulations (to 10 μs) have also been performed (see Table 1). Analysis of the interactions (see S5B Fig.) showed that the CL binding sites were retained over this time period.

Fig. 3 shows the CL interactions with arginine and/or lysine residues in the CL binding sites identified above. In the CG-MD simulations the interactions of CL molecules with UraA are mediated by interactions of the negatively charged phosphate atom of the CL headgroup with polar part of the protein and hydrophobic interactions of the acyl chains with the non-polar belt of the membrane protein. The most common residues involved in the interaction with the phosphates are arginine and lysine as has been found in other simulations [12]. In the CL binding site 1, CL interacts with R299 located in a short loop connecting TM helix 10 and 11 and/or R4 in the mobile N-terminus of the protein near the exit site of the transporter. The CL interaction site in the outer leaflet involves K321 located at the N-terminus of the short helix connecting TM helices 11 and 12 and W212 at the N-terminus of TM helix 7. Hydrophobic interactions also play a role in creating lipid binding grooves in UraA. CL tails interact with the hydrophobic part of the protein located below the arginine and lysine residues identified above. The surface of UraA contains a number of hydrophobic grooves that accommodate the lipid acyl chains. The surface created by TM helices 2, 4, 9 and 11 is quite smooth. There are hydrophobic grooves between TM helices 1 and 6, TM helices 5 and 6, and between TM helices 13 and 14 and the rest of the protein. The simulations (S3 Video) indicate that while the head groups of the lipids are immobilized by interaction with basic residues in the protein, the acyl chains are quite dynamic.

Interestingly, in all individual CG-MD simulations a high density of POPG molecules was also observed around the protein. The POPG lipids interact with the majority of the protein lipid exposed surface however basic residues form the highest number of interactions with POPG (Fig. 2, S4, S6 and S8 Figs.). This creates an acidic annulus around the protein, which may be essential in UraA function by hoarding protons required for transport. Similar annuli of anionic lipids have also been observed around TM helices [38] and around the TM domains of receptors e.g. EGFR [39]. Simulations studies have also shown that the mobility of lipids in an annulus up to ∼3 nm from the membrane surface is smaller compared to the bulk region of the bilayer [40,41]. POPE can bind indiscriminately to also form a complete annulus of lipids around the protein (S8C Fig.). All the interacting residues with the CL, POPG and the POPE lipids are shown in Fig. 2 and S6 Fig. Calculation of the mean number of lipids in contact with the protein reveals that 2 - 3 CLs and ∼ 8 POPG lipids are in contact with the protein (S7 Fig.).

To quantify the enrichment of anionic lipids around the protein we have calculated the ratio of the mean density of each lipid type in each leaflet in an annulus close to the protein and in the bulk region of the bilayer (*S*; see Methods and S4 Fig. for details of this analysis). If *S* > 1 then there is enrichment of the specific lipid type around the protein whereas if *S* < 1 it indicates that the mean density of the lipids in the bulk region is higher compared to the density of the lipids close to the protein. For POPG *S* = 1.16 for the outer leaflets and 1.33 for the inner leaflet demonstrating an enrichment of POPG around the protein. For CL, *S* = 1.14 for the inner leaflet, and 0.59 for the outer leaflet. However if we calculate *S* using only the density of the CL lipids in the CL binding sites (divided by the mean density in the bulk region) then the local value is *S* = 5 for the inner leaflet and *S* = 2.3 for the outer leaflet. This confirms the raised density of CL molecules in the discrete CL binding sites rather than the overall annulus, and thus demonstrates the preferential interaction of CL molecules with the three CL binding sites. For reference, for the zwitterionic lipid POPE *S* = 0.63 for the outer leaflet and 0.71 for the inner leaflet.

**Fig. 4:**
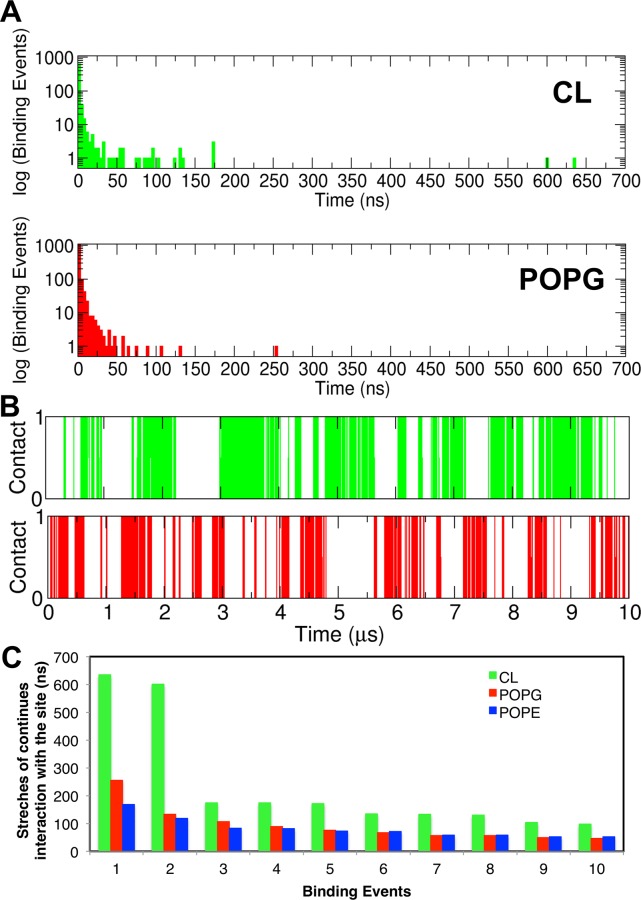
Interactions between the lipids and CL binding site 1. A. Histograms of binding event durations (defined as the time for which CL binding site 1 was continuously occupied by a lipid; see Methods for details). B. Interactions of UraA R299 residue with the POPG and CL molecules as a function of the simulation time for one of the CG simulations with the wild type UraA (10 μs simulation; UraA-CG in Table 1). An occupancy index of 1 is used when the lipids are in contact with the protein and 0 when the lipids are not in contact. See S2 Fig. for the same analysis for all residues of UraA. C. The 10 longest stretches of continuous interactions between the different lipid types and CL binding sites 1. The same analysis for the other CL binding sites is shown in S3 Fig.

To examine in more detail the association of the different lipid types with the aforementioned CL binding sites we have approximated the lifetime of interactions between the different lipids and the 3 CL binding sites (see Methods, S2 Table, Fig. 4, S2 and S3 Figs.). This analysis suggests that whilst all 3 lipid types are able to bind to the CL binding sites, the CL molecules form prolonged interactions (see S2 Table and S3 Fig.). Therefore, when a CL binding site is not occupied by CL it is likely to be occupied mainly by POPG lipids or POPE lipids to a lesser extent. Additionally, this analysis suggests that all lipid types form more stable interactions with the CL binding site 1, followed by CL binding sites 2 and 3 (S2 Table and S3B Fig.). In particular, in the CL binding site 1 CL molecules form the longest interactions, followed by POPG and POPE (S2 Table and S3B Fig.). In CL binding sites 2 and 3 the same pattern of interactions is observed however the time for which a lipid type continuously occupies a CL binding site is lower (S2 Table and S3B Fig.). Interestingly, when we calculated how many changes of single lipids of the same lipid type occurred in the CL binding site when the site is continuously occupied by a lipid type, we found that in most cases only one CL molecule interacts with the CL binding site whereas in the case of POPG and POPE lipids 3 to 7 changes of single lipids occurred in the CL binding site (S2B Fig. and S2 Table). This analysis suggests a faster exchange of the POPG and POPE lipids between the unbound and unbound state despite the fact that for this analysis we have used only those periods when a single lipid from the same lipid type occupies the CL binding site (see S2 Fig. caption). Note that the stretches of times we have obtained in our study for CL binding sites 2 and 3 are short compared to those in a recent study by Arnarez *et al*. on the interactions of CL molecules with cytochrome bc_1_ [8]. The lifetime of interactions for CL binding site 1 are comparable with the residence times at the lower occupancy CL sites of cytochrome bc_1_, but not with the times for those sites with prolonged CL interactions with the cytochrome protein. Besides the fact that we have used a different protein, the lower residence times we have observed may be due to the fact that in our study we have POPE, POPG and CL whereas in the previous study only CL and POPC lipids were present, which may induce competition between the lipids especially between the anionic CL and POPG lipids. However, the CL binding sites identified in the cytochrome bc_1_ study contain multiple lysine and arginine residues which is similar to the sites we have identified on UraA. Poyry *et al*. also studied the cytochrome bc_1_/CL interactions, using atomistic simulations, and also demonstrated that the interactions between CL lipids and cytochrome bc_1_ are mainly mediated by the interactions between the lipids and positively charged residues [42]. Other studies have shown that other anionic lipids interact with specific sites on proteins e.g. PIP_2_ lipids to ion channels [10].

We have also used the extended 10 μs simulations to examine the effect of the protein on the lateral diffusion of the POPG lipids and the CLs in the membrane (S2C Fig.). It is possible that the strong interactions between UraA and the aforementioned acidic lipids would influence the lipid diffusion rates. Indeed, the diffusion of the CL lipids in a bilayer with UraA decreases compared to a bilayer with the same lipid concentration but with no protein component (e.g. the calculated diffusion coefficient for the simulation with UraA in S2C Fig. is 2.5 ± 0.1 x10^−7^ cm^2^/s in the inner leaflet and 3 ± 0.1 x10^−7^ cm^2^/s in the outer leaflet compared to 4.5 ± 0.2 x10^−7^ cm^2^/s in the simulations without any UraA present). The diffusion of the POPG molecules is also somewhat slower in the system where the protein is present compared to a bilayer with the same lipid concentration but with no protein component (S2C Fig.), possibly due to their interactions with the protein. Interestingly, calculation of the mean diffusion coefficient for the lipids in the system without any protein component (pcpgcl-CG in S1 Table) reveals diffusion coefficients of 4.5 ± 0.2 x10^−7^ cm^2^/s for CL, 5.9 ± 0.3 x10^−7^ cm^2^/s for POPG and 6.2 ± 0.2 x10^−7^ cm^2^/s for POPE. This suggests that the four acyl chains of the CL and its larger cross-sectional diameter slow its diffusion about 1.5-fold relative to POPE or POPG. Comparison of the diffusion coefficients of POPE and POPG with those in a CG-MD study with a similar mixture of lipids (but not with the inclusion of CL lipids and with a higher number of proteins in the bilayer) suggests a somewhat lower diffusion coefficient for the POPG and POPE lipids in our system (5.9 ± 0.3 x10^−7^ cm^2^/s for POPG in our system compared to 8.5 ± 0.3 x10^−7^ cm^2^/s for POPG in Goose *et al*. [41]). Short atomistic simulations by Rog *et al*. [43] and by Murzyn *et al*. [44], again with a POPE/POPG mixture, have suggested short timescale diffusion coefficients for POPG of ∼3.5 x10^−7^ cm^2^/s. Recent microsecond scale simulations of a POPE/POPG mixture showed that the lipid diffusion coefficient is dependent on the NaCl concentration present, with a lipid lateral diffusion coefficient of 3.1 x10^−8^ cm^2^/s in the presence of 0.15 M NaCl [45].

**Fig. 5:**
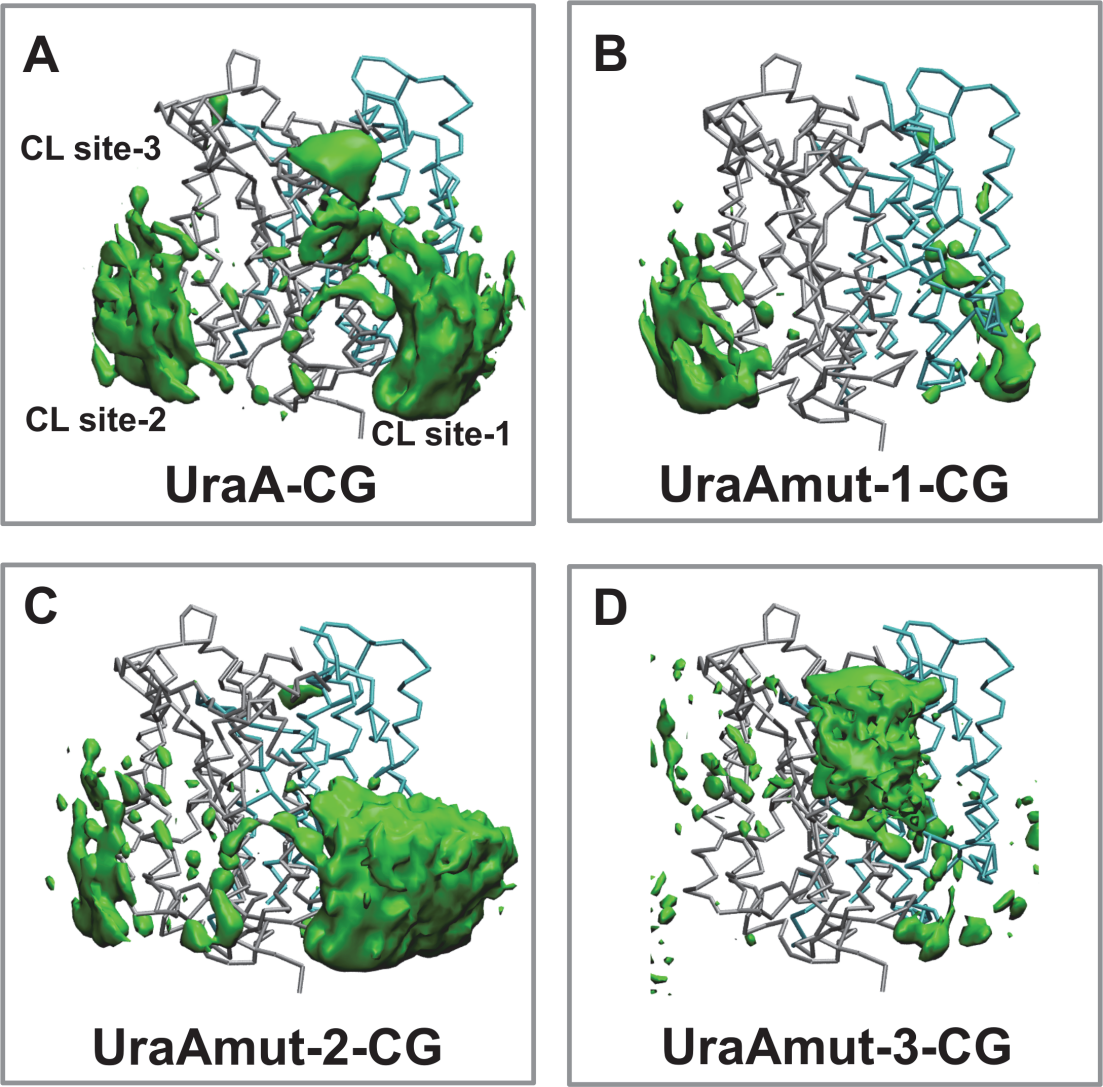
Mutations on the CL binding sites. Occupancy plots for the location of cardiolipins (CL; green) around UraA. The occupancy was calculated as the average over the multiple coarse-grained simulations of the wild type and the mutated forms of UraA (Mut 1: UraAmut-1-CG, Mut 2: UraAmut-2-CG and Mut 3: UraAmut-3-CG; see Table 1).

To test the essential role of the basic residues in UraA in CL binding and to examine whether CL molecules bind cooperatively to the three CL binding sites, three different sets of *in silico* mutations (mutating basic residues in the CL binding sites of UraA facing the outer bilayer leaflet (UraAmut-2-CG), facing the inner bilayer leaflet (UraAmut-3-CG) or a combination of the two (UraAmut-1-CG)) were performed (Table 1) and the CG-MD simulations were rerun with the mutated UraA in the lipid mixture. All the simulations with the mutated forms of UraA were initiated by replacing the wild-type protein with the mutated form of the protein after the exchange of lipids step as described in the Methods section. Fig. 5 shows the distribution of CLs in the simulations with the mutated form of the protein. In the UraAmut-1-CG system, mutation of basic residues in CL site-1 and CL site-3 (R4A and R299A in site 1 and K321A in site 3) resulted in the loss of these two CL binding sites. In this case only the non-mutated CL side-2 was observed. Similarly in the UraAmut-2-CG (K157A, R222A, K321A, K381A, K390A in site 1) and UraAmut-3-CG (R4A, R11A, R177A, R299A, R351A, R357A and R362A in site 1 and K62A, K109A, K110A, R265A and K407A in site 2) systems mutation in the CL binding site 3 and CL binding sites 1 and 2 respectively resulted in the loss of the mutated CL binding sides. The reduction of the number of CLs associated with the protein due to the mutation of the CL binding sites is also demonstrated in S7A Fig. To test perhaps more “biological” mutations we have also performed additional sets of simulations with the following *in silico* changes: (i) K321A, (ii) K109A/R265A, and (iii) R4A/R299A. In all cases the simulations resulted in the loss of binding of CL at the mutated site (S6D Fig.). In systems (i) and (ii) in addition to the strong interactions of the CL lipids with the non-mutated CL binding sites, interactions of CL molecules with R222 and K381 in (i) and R11, K62, K64 and R351 in (ii) were also observed. This augments our previous observation that basic residues in the three CL binding sides regulate the CL/UraA interactions and that CL bind to the 3 CL binding sites independently. Note that when both CL binding sides in the cytosolic part of the protein were mutated the association of the POPG lipids with UraA in the inner leaflet was also reduced (S7 Fig.). Convergence analysis of the simulations showed that all simulations were converged after 4 or 5 repeat simulations (S5C Fig.).

### Atomistic simulations of UraA

**Fig. 6:**
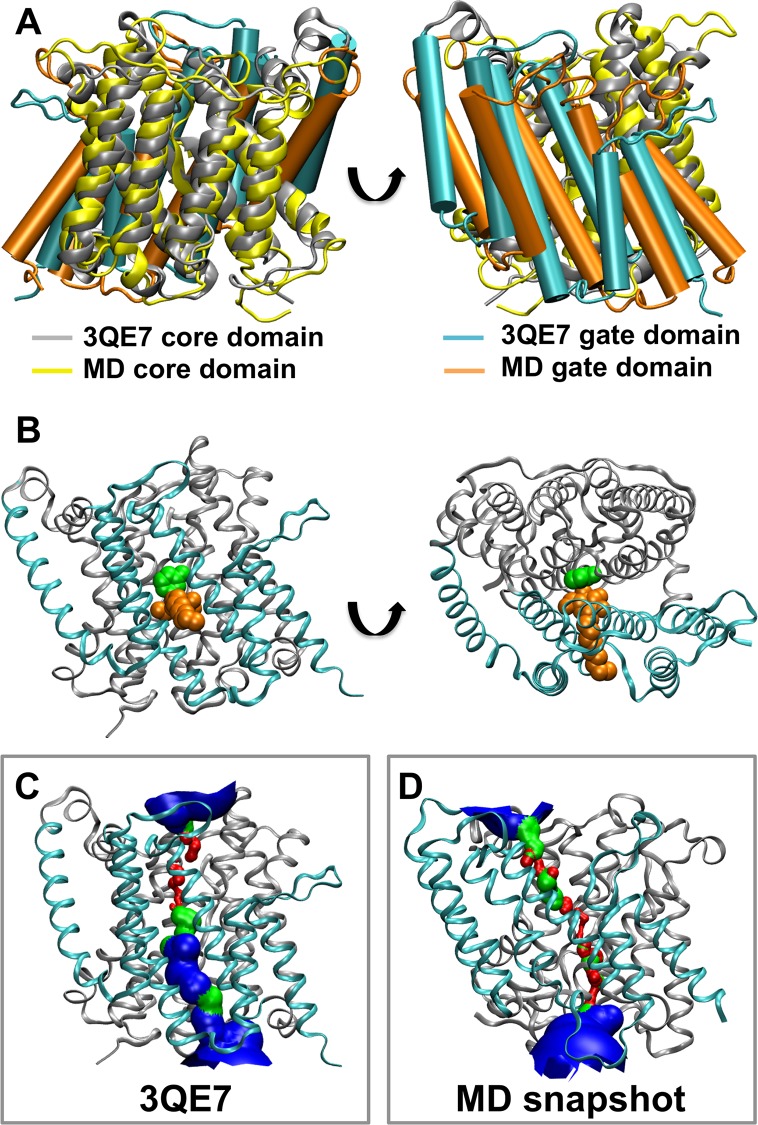
Closed state of UraA. A. Alignment of the core domain of the final snapshot of one of the atomistic simulations (UraA-AT) with the core domain of the crystal structure. B. Ribbon structure of UraA showing the location of the bound uracil (green) and NG detergent (orange). C,D. Pore lining surfaces of the proteins aligned in A. Red is used when the pore radius is lower than 1.15 Å, green when the pore radius is between 1.15 and 2.3 Å and blue when the pore radius is larger than 2.3 Å. Note the loss of water cavity (blue) from the inward-facing passage during the simulation to produce a closed conformational state of UraA.

To study the possible conformational changes within the protein and the preferential interaction of UraA with the different lipids in the bilayer the final snapshots from four of the ten repeat CG-MD simulations with the WT UraA in the POPE/POPG/CL lipid mixture were converted to an atomistic representation (see Methods for more details). This is a standard protocol used in previous studies where interactions between a protein and lipid molecules were studied. In all the atomistic snapshots CLs were bound in the aforementioned CL binding sites. Atomistic simulations (2 x 100 ns and 2 x 500 ns) were then run. Calculation of the root mean square displacement (RMSD) of the protein during the simulation revealed a sharp increase of the protein RMSD at the beginning of the simulations to ∼0.35 nm. The RMSD for the longer AT-MD simulations subsequently increased to final values of ∼0.5 nm (S9A Fig.). This increase is due to large fluctuations of the unstructured regions of UraA. Indeed, calculation of the route mean square fluctuation (RMSF) showed that core secondary structure elements have RMSFs between ∼0.1 and ∼0.2 nm whereas unstructured regions have RMSFs between ∼0.4 to ∼0.6 nm. Alignment of the crystal structure with the final snapshot of the extended simulations demonstrates that the integrity of all 14 transmembrane helices was maintained during the simulation, however the positions and angles of the helices that form the gate domain changed from the crystal structure (Fig. 6 and S9 Fig.). Calculation of the transport path of the protein at the end of all atomistic simulations, using the program HOLE, revealed that the protein moved from an inward facing conformation that initially bound uracil (and a single detergent) to a closed conformation collapsing the water-filled passage to the cytosol (Fig. 6 and S9 Fig.). This is presumably due to the fact that the simulations were performed in the absence of uracil. Alignment of the gate and the core domains from the end of the four atomistic simulations with the crystal structure reveals no significant conformational changes within the domains themselves (S9G,H Fig.). This suggests that in the absence of the uracil molecule a rigid body movement of the cytosolic part of the gate domain toward the cytosolic part of the core domain shifts the protein conformation to a close state. Simulations of the UraA protein in a simpler POPC or POPE lipid bilayer resulted in the same shift of the protein from the inward facing to a closed conformation (S9 Fig.), showing that the conformation change was not dependent upon a specific lipid composition. During the simulation no ion flux is observed. However occasionally we observe a small flux of water molecules.

Closer examination of the interactions between CL and the protein at the atomistic level suggests stable interactions between the CL oxygen atoms and the protein for the complete duration of the simulations (Fig. 3 and S10 Fig.). The contacts made between UraA and CL in the corresponding CG-MD simulations were maintained during the atomistic simulations. In particular CL head groups can be seen to form significant interactions with the R4 and K299 residues in CL binding site 1, K109 and R265 residues in the CL binding site 2 and K321 residues in CL binding site 3 (Fig. 3). The CL tails form dynamic interactions with the hydrophobic part of the CL binding sites. Additionally, the annulus of the CL and POPG lipids around the proteins is retained on the timescale of our AT-MD simulations.

## Discussion

### Cardiolipin binding sites in UraA

The major finding of our study is the identification of three CL binding sites in UraA. That these were not seen in the crystal structure is perhaps not surprising given that the purification/crystallization procedures extracts the majority of any bound lipids and that CL is a relatively minor (5%) component of the *E*. *coli* inner membrane. Indeed, no phospholipids could be detected in purified samples (∼1 mg protein/ml) of His-tagged UraA prepared using the detergent dodecylmaltoside and Ni-affinity chromatography. CL has some unique physiochemical properties [46]. It consist of two phosphatidic acid linked together by a central glycerol. The free hydroxyl on the glycerol can form a hydrogen bond with the neighboring phosphate oxygens. This results in the formation of an acid-anion and a CL molecule with a single net negative charge with one phosphate having a typical low pKa (∼2–3) and the second phosphate having a much higher pKa in the physiological pH range [47]. CL can thus act as a ready proton acceptor and donor. CL also has a very small head-group compared to the size of its hydrophobic region with four acyl chains. This allows CL molecules to pack closely together to form clusters (e.g. CL “lipid rafts”) in mitochondrial and bacterial membranes. The cross-sectional area of CL is decreased upon binding of counter-ions like sodium due to reduction in electrostatic repulsion. The CL binding site identified in the extracellular site of UraA is located close to the transport path implying the CL may be important for providing the proton required for transport, as suggested for other proton transporters [8,48]. The inner CL binding sites may also have active role in the proton transport, by buffering protons as they exit the protein, however further investigation is required to identify the exact role of these CL binding sites in transport. Calculation of the binding energy of CL in cytochrome c oxidase by Marrink *et al*. using extended CG-MD simulations (100 μs) revealed a free energy of association of ∼-30 kJ/mol for the main CL binding sites [9]. A quantitative measure of the association of lipids with specific membrane proteins may be given by EPR and fluorescence quenching methods [49]. Note that no associated lipids were reported in the crystal structure of UraA [20]. Other techniques such as crosslinking often require the use of chemically-modified lipids that have different properties from native lipids used in this study. The residues which are involved in the UraA/CL interactions are expected to be exposed to the lipid environment when the protein is in the outward open state and thus it is possible that CLs also associate with UraA when the protein is in the outward open conformation. However structural data for UraA in the outward open state are needed to be able to study this computationally.

UraA is a member of the nucelobase/ascorbate transporter (NAT) family, of which there are 10 family members in *E*. *coli*. We have shown that certain arginine and lysine residues in UraA are required for CL binding. How conserved are these residues in the NAT family in *E*. *coli*? Alignment of all 10 members of the NAT family in *E*. *coli* suggests that an arginine residue at the UraA R4 position is conserved in 4 out 10 members of the family. However with the exception of YbbY all other members of the family have a conserved basic residue in the vicinity of R4 (6 residues away; S11 Fig.). Inspection of UraA suggests that this conserved positive residue is also located in the CL binding site 1 described in this study. Additionally, all the members of the NAT family found in *E*. *coli* have a basic residue in the same position as R299 or in the same loop 1–3 residues distally (S11 Fig.). Interestingly, residue R299 is found at the end of the NAT-signature motif. This is a motif which is conserved in the NAT/NC2 family of proteins ([Q/E/P-N-x-G-x-x-x-x-[RKG]) and it was shown to be important in the function of UapA [50]. In particular R299 corresponds to R417 on UapA, the fungal uric acid/xanthine permease from *Aspergillus*, and mutation of this residue affect the kinetics and specificity of UapA for uric acid [50]. Additionally, cysteine scanning mutagenesis of the xanthine permease XanQ (YgfO) residue G333, which again corresponds to the UraA R299 residue (S11 Fig.), suggests that it plays a crucial role to the specificity of XanQ for different substrates [51]. Furthermore, replacement of certain basic residues located within TM segments in XanQ (K164, K249, R341, R385) with cysteine resulted in functional transporters but with significant decreases in xanthine uptake [52]. Mutation of basic residues (R285, R286, R296) in the cytosolic loop connected TM 8 and 9 showed modest decreases in transport [53]. The K298C mutant showed ∼50% diminished initial xanthine uptake. K298 is located at an equivalent position one residue proximal to R265 in UraA at the beginning of TM9, while R285/286 in XanQ are lysine residues in UraA.

In CL binding site 2 a basic residue in the same position as R265 is present with the exception of UapA, XanQ, XanP and PurP (S11 Fig.). Note, however, that XanQ, XanP and PurP proteins have an adjacent positive residue to the R265 position whereas UapA has a basic amino acid 3 residues distal from R265. K109 is poorly conserved in the *E*. *coli* NAT/NCS2 family, however basic residues are located in the same loop 3–5 residues distally in all family members. Finally, K321 is conserved only in some family members (RutG, UacT) but not others. CL is not found in eukaryotic plasma membranes and thus key basic residues responsible for CL interaction in bacteria are not expected to be conserved in these NAT family members.

CL plays an essential role in the assembly and functioning of mitochondrial and bacterial membranes [54]. Yeast mutants deficient in CL biosynthesis have impaired mitochondrial function [55]. The ADP/ATP translocator is associated with CL clusters in the inner mitochondrial membrane along with respiratory sub-complexes [56] and this transporter is dependent upon CL for optimal activity [57]. The mitochondrial enzyme creatine kinase induces clustering of CL [58] and in turn, CL promotes the association of ATP synthase into ribbon-like structures in the mitochondrial membrane [59].

CL exists in clusters in bacterial membranes in regions of high curvature such as in “mini-cells” [60] and at the cell poles [61,62]. ProP, a H^+^-proline symporter, is localized to CL clusters at the cell poles of *E*. *coli* [63]. Phospholipids interact with membrane proteins during their insertion into the membrane acting as chaperones to assist in their folding [64]. Indeed CL interacts tightly with the bacterial SecYEG translocon where it creates a high-affinity binding site for the SecA ATPase and localizes the complex to the cell poles [65]. This suggests that CL may be involved in membrane protein folding and insertion into the bilayer during biosynthesis. CL may also act as a “proton trap” through its acid-anion structure and is commonly associated with proteins involved in oxidative phosphorylation, associating with sub-complexes and providing a source of protons for the ATP synthase [66]. CL is found associated with sub-complexes of the respiratory chain such as in the bacterial photoreaction center [67,68] and the yeast cytochrome bc1 complex [6,69]. The association of CL with proton-driven symporters like UraA may point to a similar role by providing a source of buffered protons in the vicinity of these transporters. As in the case of ProP, CL may also provide a mechanism to cluster and localize proton-driven transporters to the cell poles in bacteria. Proton may be channeled directly between CL and the proton-binding site in the center of the transport protein via a proton highway composed in part of acidic residues. For example, CL is located close to the exit site for protons in cytochrome oxidase [66]. A similar role of CL has been also identified recently for the cytochrome c oxidases [9].

### A closed state for UraA

The second major finding of this study is the observation of a closed state of UraA, resulting from removal of the substrate uracil and a stabilizing non-native detergent molecule. Membrane proteins in general can tolerate changes in membrane lipid composition, which occur commonly in micro-organism as they adapt to changes in their environment [2]. Any changes in conformation that occurred during the simulation may be due to lipid interactions or loss of the substrate, or a combination of both factors. We found a similar change in the UraA structure in three different lipid environments (POPC, POPE and the lipid mixture), suggesting that lipids did not greatly influence the protein motions in moving from an empty to a closed state conformation. This suggests that the CL binding is not required for the transition from the inward facing to the closed state.

There is a water-filled passage leading from the bound uracil to the cytosol, partly occluded by the bound detergent. The protein can respond in two ways: 1, by filling in the space with water or 2, by changing conformation to close the space. The final atomistic structure of UraA in a bilayer is in a closed state conformation. This change occurred regardless of the lipid composition and thus is due to performing the simulations with the empty carrier. Thus, the MD simulation produced a closed state of the carrier, i.e. a state that may not be readily accessible by crystallography or other structural methods and that is an intermediate in the transport mechanism.

### Membrane proteins in lipid bilayers

Molecular dynamics simulations provide a powerful tool to analyze the structure and dynamics of membrane proteins in lipid bilayers of defined composition [4]. A molecular dynamics simulation of LacY, in various lipids identified specific interactions between the lipid head-groups and sites on the protein [70]. Recent studies also identified cardiolipin (CL) binding sites in the cytochrome bc_1_ transporter [8] and in the cytochrome c oxidase [9] using CG-MD simulations. Similar studies identified specific interactions between cholesterol [15] or PIP lipids [10,11] with integral membrane proteins. The inner membrane of gram-negative bacteria, like *E*. *coli*, is composed of approximately 75% PE, 20% PG and 5% CL under normal laboratory growth conditions at 37°C [71]. In this simulation we found an annulus of anionic lipids around the UraA protein, followed by concentric rings spaced ∼5 Å apart defined by the cross-sectional diameter of the lipid. Rings are discernable out to 20 Å from the protein surface. This suggests that the protein restricts the mobility of the annular lipids, a property of membrane proteins noted for some time [72,73]. The flexible acyl chains can fill in gaps in the protein’s hydrophobic surface and the head groups can mediate more specific interactions with the polar parts of the protein [74,75]. Molecular simulations indicate that the association of annular lipids, with aquaporin for example can be quite dynamic [12]. Tightly-bound lipids have been identified in a number of X-ray crystal structures of membrane proteins showing that there are specific binding sites for lipids on the surface of some membrane proteins, which may assist in their folding or functioning [7]. These interactions involve tight binding of the acyl chains to the hydrophobic surface of the protein as well as polar/ionic interactions involving the head groups. The different simulations run for UraA indicate that CL is preferentially associated with the protein, although this lipid was only present at a 5% content. Indeed, positively-charged residues on hydrophilic loops of the protein can engage anionic lipids in a dynamic fashion, the lipids moving on and off the protein over the overall ∼40 μs time course of the simulation. PE although in excess does not interact in a stable manner with UraA, likely due to its zwitterionic nature. The dynamic nature of the lipid-protein interactions observed with UraA may allow the protein to adapt to different lipid environments that the organisms like *E*. *coli* might encounter [2].

### Limitations

It is important to consider possible limitations of simulations discussed in this study. The use of a CG-MD based approach implies some approximations in the protein and in the lipids. It has been shown, however, that CG-MD simulations correctly predict interactions of CL [8] and of other lipids (e.g. PIP_2_) with integral membrane proteins [10–15]. Conversion of the final snapshot of four individual CG-MD simulation to a fully atomistic representation and extended AT-MD simulation runs confirm the stability of the identified CL binding sites (S10 Fig.) and gives more detail information about the positioning of the CL lipid tails. The use of an elastic network model within the CG-MD simulations restricts any possible conformational changes of the protein. The use of a serial multiscale approach, with subsequent atomistic simulations, in part addresses this limitation allowing us to observe a closed state of the protein. Despite seen some conformational changes within the protein more extended simulations and/or exchange sampling techniques (e.g., dynamic importance sampling (DIMS) [76] or self-guided Langevin dynamics [77] are likely to be needed to more fully address possible conformational changes (e.g. outward facing conformation). Another limitation which arises due to the intrinsic approximations of the CG representation is that quantities such as the durations of protein/lipid interactions or diffusion coefficients of lipids are likely to be influenced by the coarse-graining of the protein and to be sensitive to the exact protocol used. Such analyses, however, provide a semi-quantitative measure of the stability of protein-lipid interactions observed in the CG-MD simulations. The removal of the uracil from the binding site produced a novel closed state of UraA, which may form part of the transport cycle. In future studies it will be important both to include the uracil molecule and possibly extend the time frames to capture the binding and movement of substrate across the membrane and the various conformational state of the transport protein in real time. It will also of course be important to test e.g. the predictions concerning CL binding sites and key residues via experimental mutagenesis studies.

## Supporting Information

S1 Fig Flowchart of the simulations performed for this study. Schematic representation of the inputs (white background boxes) and outputs (grey background boxes) of the simulations performed. See Methods for further information.
(TIF)

S2 Fig Lipid mobility and interactions with UraA. A. Interactions of UraA with the POPE, POPG and CL molecules as a function of the simulation time for one of the CG simulations with the wild type UraA (10 μs simulation; UraA-CG in Table 1). Yellow is used when the lipids are in contact with the protein and black when the lipids are not in contact. B. Total number of changes of single lipids associated with CL binding site 1 during the longest stretches of continuous interactions between the different lipid types and CL binding site 1 (sim2 in S2 Table). For this analysis we have used only those periods of time during which the CL binding sites were occupied by a single lipid of the same type. For all lipids there were frames during the simulations for which the binding site was occupied by more than one lipid of the same type. These frames were excluded from our calculation. Thus we consider a change from a single lipid to another lipid only when the CL site is occupied by a single lipid and this lipid is replaced by a single lipid of the same type. S2B Fig. and S2 Table show the total number of the aforementioned exchanges during the longest stretches of continuous interactions between the different lipid types and CL binding sites. Overall for sim2, for which we show the results, ∼75% of the time a single CL lipid occupied the site and ∼25% of the time more than one CL lipids associated with the CL binding site 1 when CL site 1 was occupied by CL lipids (using a 1.1 nm cutoff distance to define contact; see Methods). CL site 2 was occupied ∼85% by a single CL lipid and CL site 3 was occupied ∼90% by a single CL lipid. The same analysis showed that when the CL sites were occupied by POPG lipids, CL site 1 was occupied ∼67% of the time by a single POPG lipid, CL site 2 was occupied ∼70% of the time by a single POPG lipid and CL site 3 was occupied by a single POPG lipids ∼65% of the time. For the rest of the time more than one POPG lipids associated with the CL binding sites. When the sites were occupied by POPE lipids CL site 1 was occupied ∼50% of the time by a single POPE lipid, CL site 2 was occupied ∼65% of the time by a single POPE lipid and CL site 3 was occupied ∼60% of the time by a single POPE lipid. The interaction pattern is broadly similar for the other two simulations. During the longest stretches of continuous interactions the percentage of the single CL lipids which occupy the CL sites are either slightly higher or the same as the overall percentage (see above). In contrast in the case of POPG or POPE lipids the percentage of single lipids which occupy the CL sites during the longest stretches of continuous interactions is significantly lower compared to the overall percentages and in some cases the CL sites are occupied for up to ∼80% of the time with more than one POPG or POPE lipids. Therefore using only the frames which a single lipid occupies the CL binding sites is a rather good approximation for calculating how many different CL lipids continuously occupy a CL binding site but for the POPG and POPE lipids we use a rather small subset of the interaction between the aforementioned lipids and the CL binding sites. We have decided, however, to use the same protocol for these lipids since this provides a rough measure of the faster exchange of these lipids compared to the CL molecules. C. Mean square displacement (MSD) relative to the time for the POPE, POPG and the CLs for the pcpgcl-CG (A) and UraA-CG (B) systems (see Table 1 and S1 Table).
(TIF)

S3 Fig Lifetime of interactions between UraA CL binding sites and the different lipids. A. Histograms of the binding events observed during the simulations relatively to the time for which a CL binding site was continuously occupied for the 3 different lipid species in our simulations. B. The 20 highest stretches of continuous interactions between the different lipid types and the different CL binding sites are shown. See Methods on how this analysis was performed.
(TIF)

S4 Fig Lipid density around UraA. A Mean density of CL, POPG and POPE lipids around UraA. The density was calculated by fitting the protein to a reference structure and merging all the trajectories from the three extended CG-MD simulations. B. Scatter plots with the density of POPG lipids shown in A for the inner and the outer leaflet of the bilayer. The areas of the density plots (close to the protein in red and bulk region of the bilayer in blue) used to calculate the ratio S (see Methods for more details) are also shown.
(TIF)

S5 Fig Electrostatic profile of UraA. The black lines indicate the bilayer phosphate atoms. Blue indicates a positive surface and red a negative surface. The electrostatic calculation was performed using APBS [78] in PyMol [79]. Note that the basic cluster on cytosolic side of UraA is consistent with the “inside-positive” rule [80]. B,C. Convergence analysis. Occupancy of the CL molecules (green) in the simulations with the WT and a mutated form of the protein is shown by using 4, 8 and 10 repeat simulations. The systems used were chosen randomly. This analysis suggests that the simulation system converges after 4 or 5 repeat simulations.
(TIF)

S6 Fig Interactions of UraA with the lipids. A,B,C. Normalized average number of contacts for the CG-MD simulations with the WT UraA. A cut-off distance of 7 Å for POPE and POPG and 8 Å for the CLs due to their largest headgroup was used. For the normalization, the number of contacts of a residue with a lipid type was divided by the largest number of contacts that the same lipid type made with any residue in the protein. This means that the residue with the most frequent contacts with a lipid type will have the value of 1 and the residue with the lowest contacts (or with no contacts) with a lipid type will have the value of 0. D. Occupancy plots showing the relative probability of occurrence of cardiolipin (CL; green), around UraA in the simulations with the following *in silico* mutations: (i) K321A, (ii) K109A/R265A, and (iii) R4A/R299A. The Cα atom of the mutated residues in each case is shown in van der Waals format.
(TIF)

S7 Fig Number of lipids interacting with UraA. Number of CL (A), POPG (B) and POPE (C) lipids in contact with the UraA over the course of the simulations with the wild type and the mutated forms of UraA. The four different colors represent the four different simulation systems (UraA-CG in blue, UraAmut1-CG in red, UraAmut2-CG in green, UraAmut3-CG in orange; see Table 1). On average, UraA associates with ∼2 or 3 CLs, ∼8 POPGs and ∼12 POPEs in the simulations with the wild type protein. The number of CLs associated with the protein reduced to 1 or 2 in the simulations with the mutated forms
(TIF)

S8 Fig Lipid occupancy plots around UraA. Occupancy plots showing the relative probability of occurrence of cardiolipin (CL; green), of the POPG (red) and of the POPE (blue) lipids around UraA. The occupancy was calculated as the average over all repeat coarse-grained simulations of the wild type UraA (UraA-CG in Table 1). Three different views are shown: a side view (left) showing the three CL binding sites, a cytosolic view facing the inner leaflet (middle) showing CL sites 1 and 2, and an extracellular view facing the outer leaflet of the bilayer (right) showing CL site 3.
(TIF)

S9 Fig Root mean square deviation (RMSD) and the closed state of UraA. A,B,C. Root mean square deviation (RMSD) of UraA as a function of the simulation time for the atomistic systems (UraA-AT in PE, PG, CL, UraA-pc-AT in PC and UraA-pe-AT in PE in Table 1 and S1 Table). The RMSDs for the repeat simulations for each system are shown in different colors. D,E,F. Pore lining surfaces along the transport axis of the structures at the end of all the atomistic simulations (UraA-AT in D, UraA-pe-AT in E and UraA-pc-AT in F). The dotted line shows the uracil binding site as shown by *Lu et al*. [20] crystal structure. The profile was calculated using the program HOLE. The profile for each of the repeat simulations is shown in different colors. In all cases in the region between 0 to -5 nm along the transport axis the pore is closed (cytosolic side of the protein). After that region there is some variation in the profiles due to the present of more dynamic regions of the protein (e.g. dynamic loops of UraA and the end of the TM helices). The pore lining surface of the crystal structure is shown in black. G,H. Alignment of the gate (G) and core (H) domains from the end of the two extended simulations of the UraA-AT system with the corresponding domain in the crystal structure of UraA. Each simulation is shown in different color. The gate and the core domain from the crystal structure are shown in cyan.
(TIF)

S10 Fig Interactions of UraA with CLs in the atomistic simulations. A. Interactions of UraA with CLs as a function of the simulation time for all the atomistic simulations with the wild type UraA (UraA-AT in Table 1). Yellow is used when the lipids are in contact with the protein and black when the lipids are not in contact. CL exhibit a prolonged interaction with specific residues in UraA (yellow). The positively charged residues identified in the CG-MD simulations to coordinate the CL binding to the three CL binding sites are also shown (see Fig. 3 in the main text). B. Distance between the residue 350 of the UraA crystal and the same residue of UraA in the atomistic simulations relative to time. To calculate the timescale of the changes in the position of the gate domain during the AT-MD simulation we have fitted the core domain of UraA in the extended atomistic simulations to the core domain of UraA crystal structure. Subsequently we have calculated the distance of residue 350 (Cα atom) of the crystal structure to the same residue in the simulation snapshots for all simulation frames. This residue was selected because is located on the cytosolic part of helix 12 (at the end of the helical region and close to the transportation path) and its movement would give a representative timescale for the movement of the cytosolic part of the gate domain relative to the crystal structure. This distance is shown schematically in the inset picture at the bottom right of the plot. The other inset picture shows the aforementioned distance for the first 5 ns of the two extended atomistic simulations. Note that the distance at the beginning of the atomistic simulations is not 0 because an initial movement of the gate domain relative to the core domain was observed during the CG-MD simulations. C. Number of H-bonds between the different lipids and UraA for one of the extended AT-MD simulations.
(TIF)

S11 Fig Sequence alignment of UraA from *E. coli* and UapA from Aspergillus nidulans with the other 9 *E. coli* members of the NAT family. A. The basic residues on the CL binding sites are highlighted as boxes. The alignment was made using Clustal program in Jalview. The sequences are colored based on the sequence conservation using the ClystalX color scheme.
(TIF)

S1 Table Summary of all other simulations. (DOCX)

S2 Table Lifetime of the interactions between the lipids and the 3 CL binding sites. (DOCX)

S1 Video Self-assembly of POPC lipids around the UraA transporter. In this 100 ns CG-MD simulation the protein Cα atoms were restrained but the side chains were free to rotate and move. The POPC lipids are shown in bond format and the protein in VDW format. The lipid bilayer assembled around the protein very rapidly (after the first ∼15 ns of the simulation), confirming that UraA contains a hydrophobic belt occupied by detergent in the crystal structure.
(MP4)

S2 Video Occupancy analysis for the cardiolipin (CL; green) around UraA. The occupancy was calculated as the average occupancy over all repeat simulations of the wild type UraA (UraA-CG in Table 1). The UraA backbone particles are shown in bond format.
(MP4)

S3 Video Lipid binding to CL binding site 1. MD simulation showing the diffusion of two single CL molecules (green) and a POPG molecule (red) in a lipid bilayer and their binding to the CL site 1 on UraA (shown using the residues R4 and R299). Note that in the video UraA is centered in the bilayer for clarity and a smoothing of the trajectory every 10 frames was performed. In the simulation the protein is free to diffuse.
(MP4)
