# Supplementary figures and images for: Molecular Dynamics Simulations of the Bacterial UraA H+-Uracil Symporter in Lipid Bilayers Reveal a Closed State and a Selective Interaction with Cardiolipin
Source: PLoS Comput Biol. 2015 Mar 2;11(3):e1004123. doi: 10.1371/journal.pcbi.1004123 (PMC4346270; doi:10.1371/journal.pcbi.1004123)

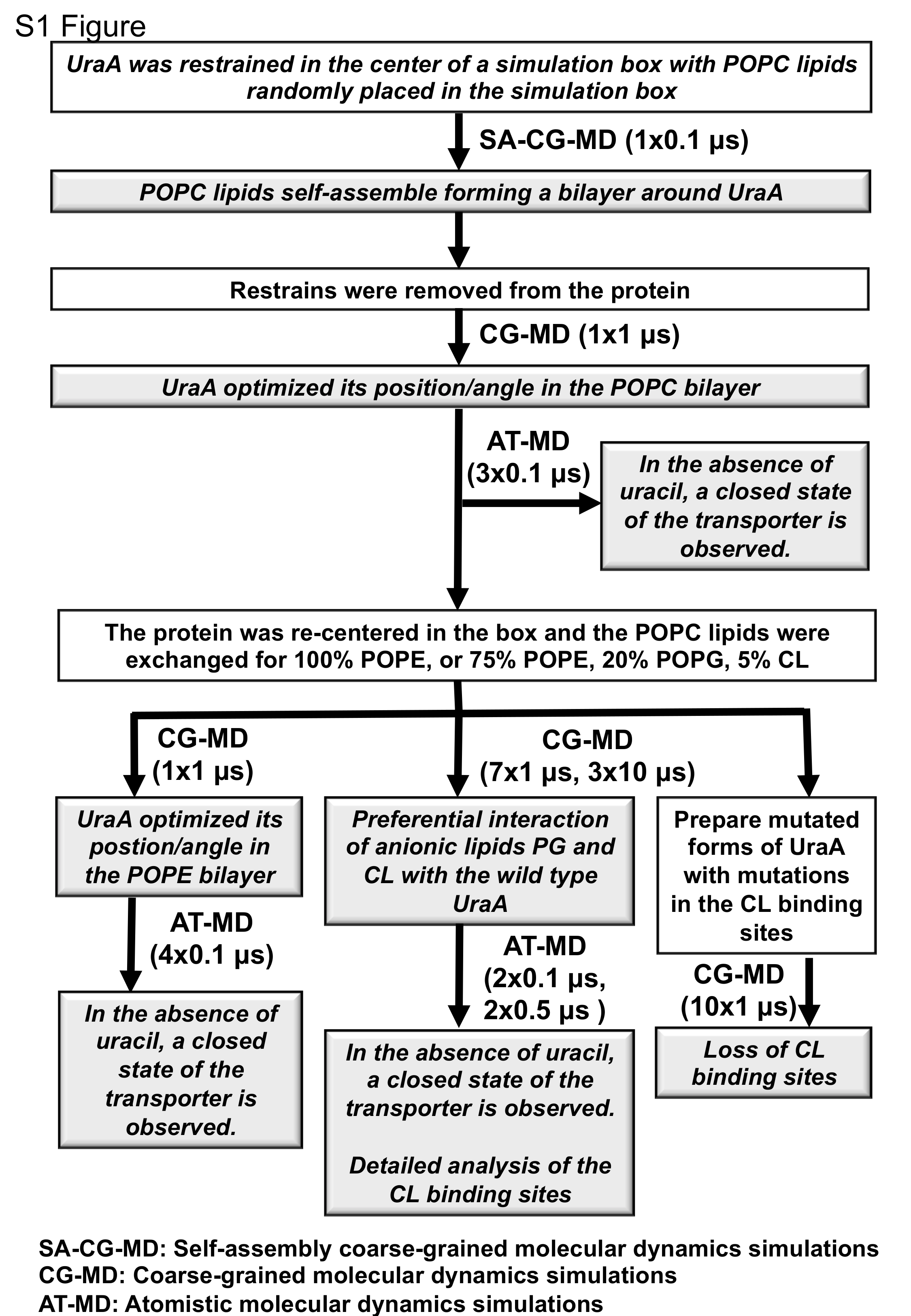

Supplement: S1 Fig — Schematic representation of the inputs (white background boxes) and outputs (grey background boxes) of the simulations performed. See Methods for further information. (TIF) [file pcbi.1004123.s001.tif]

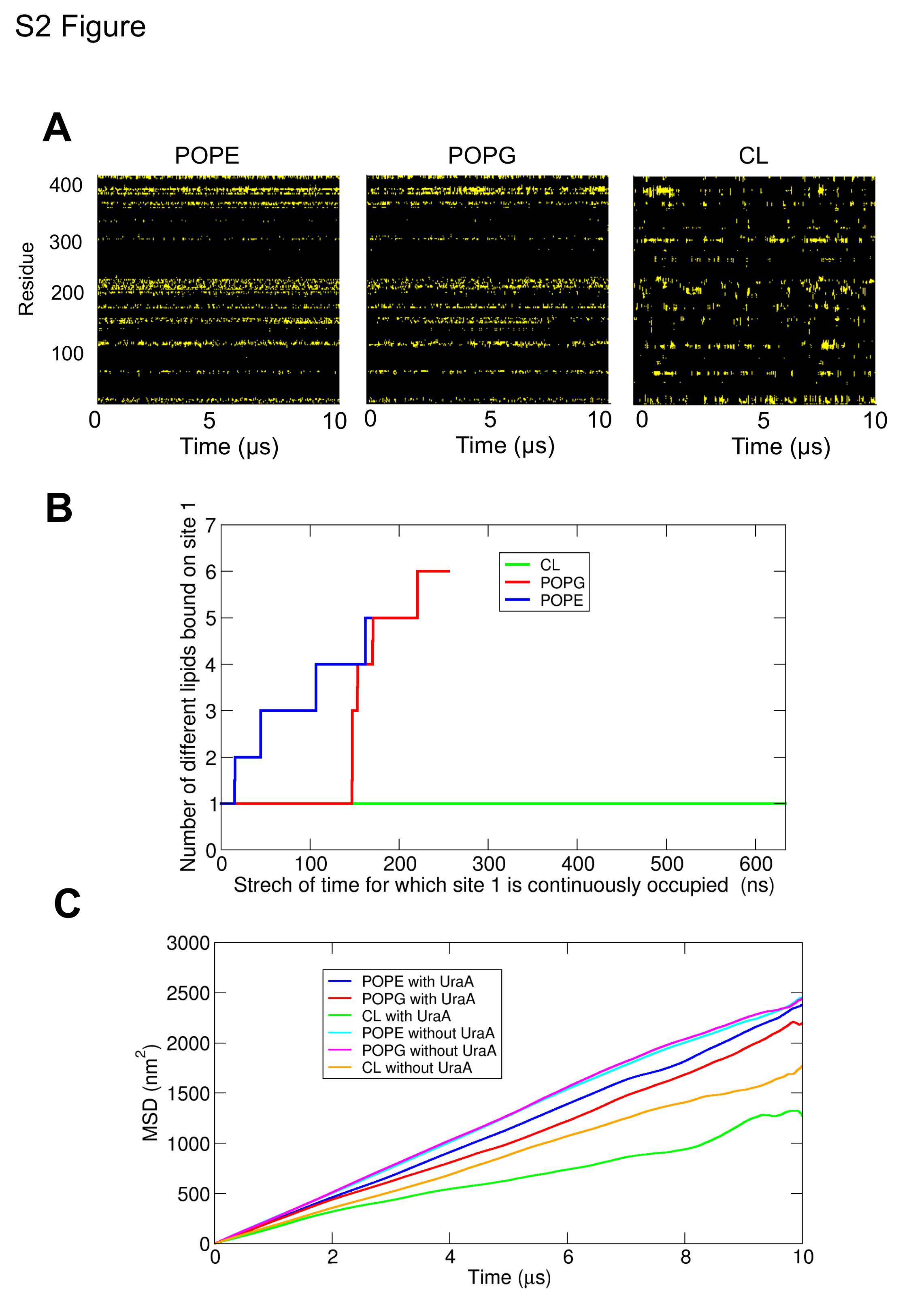

Supplement: S2 Fig — A. Interactions of UraA with the POPE, POPG and CL molecules as a function of the simulation time for one of the CG simulations with the wild type UraA (10 μs simulation; UraA-CG in Table 1). Yellow is used when the lipids are in contact with the protein and black when the lipids are not in contact. B. Total number of changes of single lipids associated with CL binding site 1 during the longest stretches of continuous interactions between the different lipid types and CL binding site 1 (sim2 in S2 Table). For this analysis we have used only those periods of time during which the CL binding sites were occupied by a single lipid of the same type. For all lipids there were frames during the simulations for which the binding site was occupied by more than one lipid of the same type. These frames were excluded from our calculation. Thus we consider a change from a single lipid to another lipid only when the CL site is occupied by a single lipid and this lipid is replaced by a single lipid of the same type. S2B Fig. and S2 Table show the total number of the aforementioned exchanges during the longest stretches of continuous interactions between the different lipid types and CL binding sites. Overall for sim2, for which we show the results, ∼75% of the time a single CL lipid occupied the site and ∼25% of the time more than one CL lipids associated with the CL binding site 1 when CL site 1 was occupied by CL lipids (using a 1.1 nm cutoff distance to define contact; see Methods). CL site 2 was occupied ∼85% by a single CL lipid and CL site 3 was occupied ∼90% by a single CL lipid. The same analysis showed that when the CL sites were occupied by POPG lipids, CL site 1 was occupied ∼67% of the time by a single POPG lipid, CL site 2 was occupied ∼70% of the time by a single POPG lipid and CL site 3 was occupied by a single POPG lipids ∼65% of the time. For the rest of the time more than one POPG lipids associated with the CL binding sites. When the sites were occupied by POPE l [file pcbi.1004123.s002.tif]

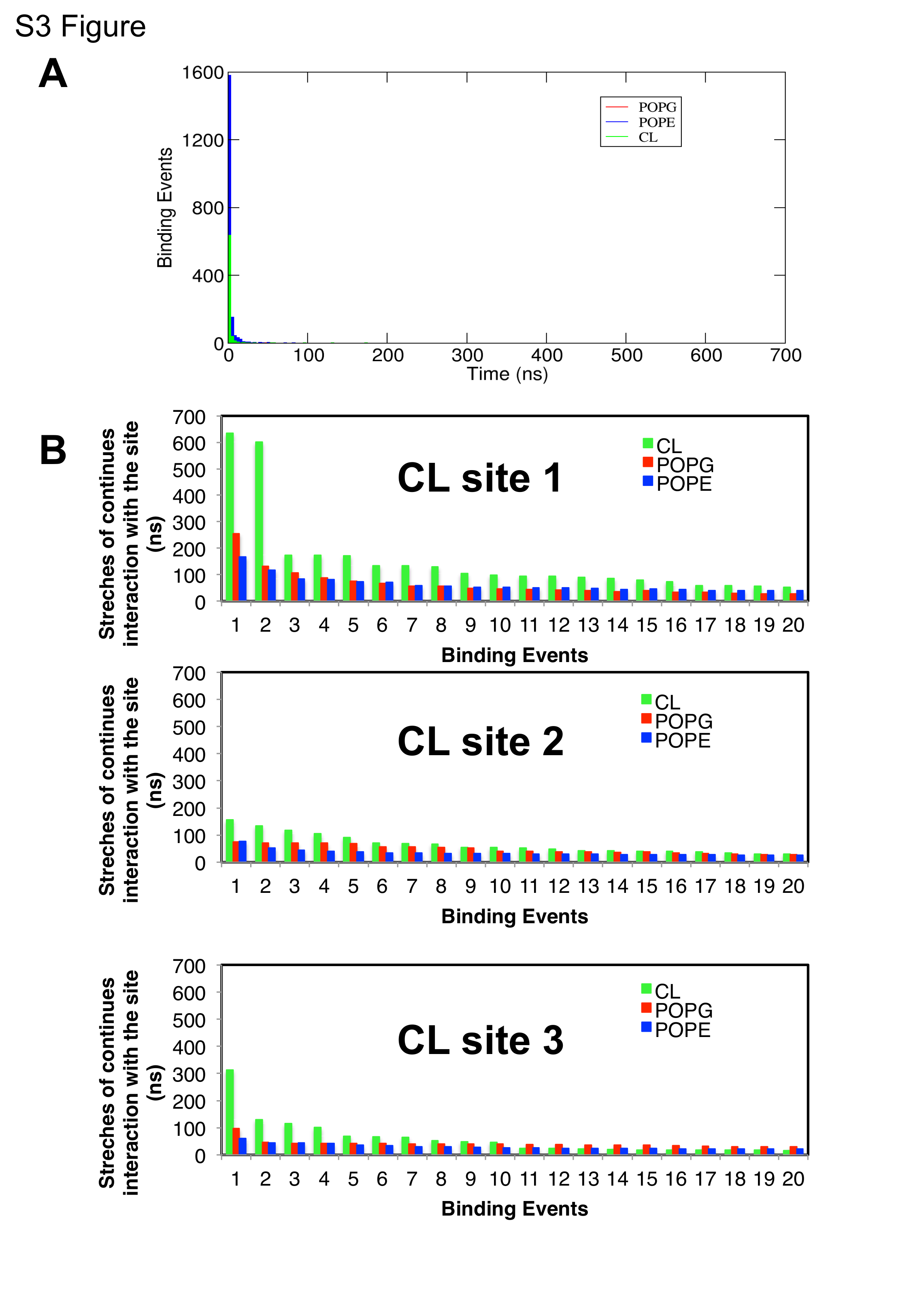

Supplement: S3 Fig — A. Histograms of the binding events observed during the simulations relatively to the time for which a CL binding site was continuously occupied for the 3 different lipid species in our simulations. B. The 20 highest stretches of continuous interactions between the different lipid types and the different CL binding sites are shown. See Methods on how this analysis was performed. (TIF) [file pcbi.1004123.s003.tif]

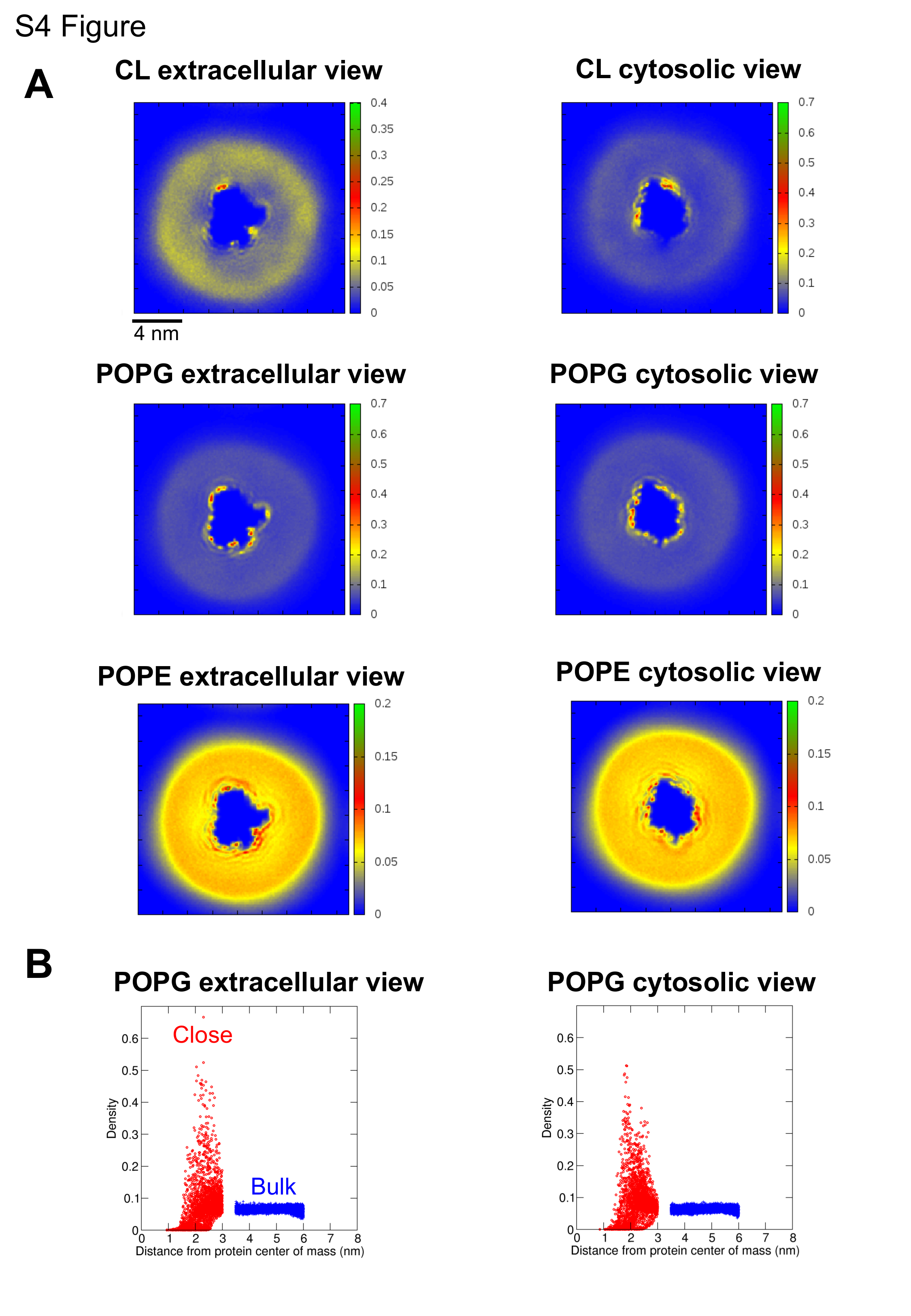

Supplement: S4 Fig — A Mean density of CL, POPG and POPE lipids around UraA. The density was calculated by fitting the protein to a reference structure and merging all the trajectories from the three extended CG-MD simulations. B. Scatter plots with the density of POPG lipids shown in A for the inner and the outer leaflet of the bilayer. The areas of the density plots (close to the protein in red and bulk region of the bilayer in blue) used to calculate the ratio S (see Methods for more details) are also shown. (TIF) [file pcbi.1004123.s004.tif]

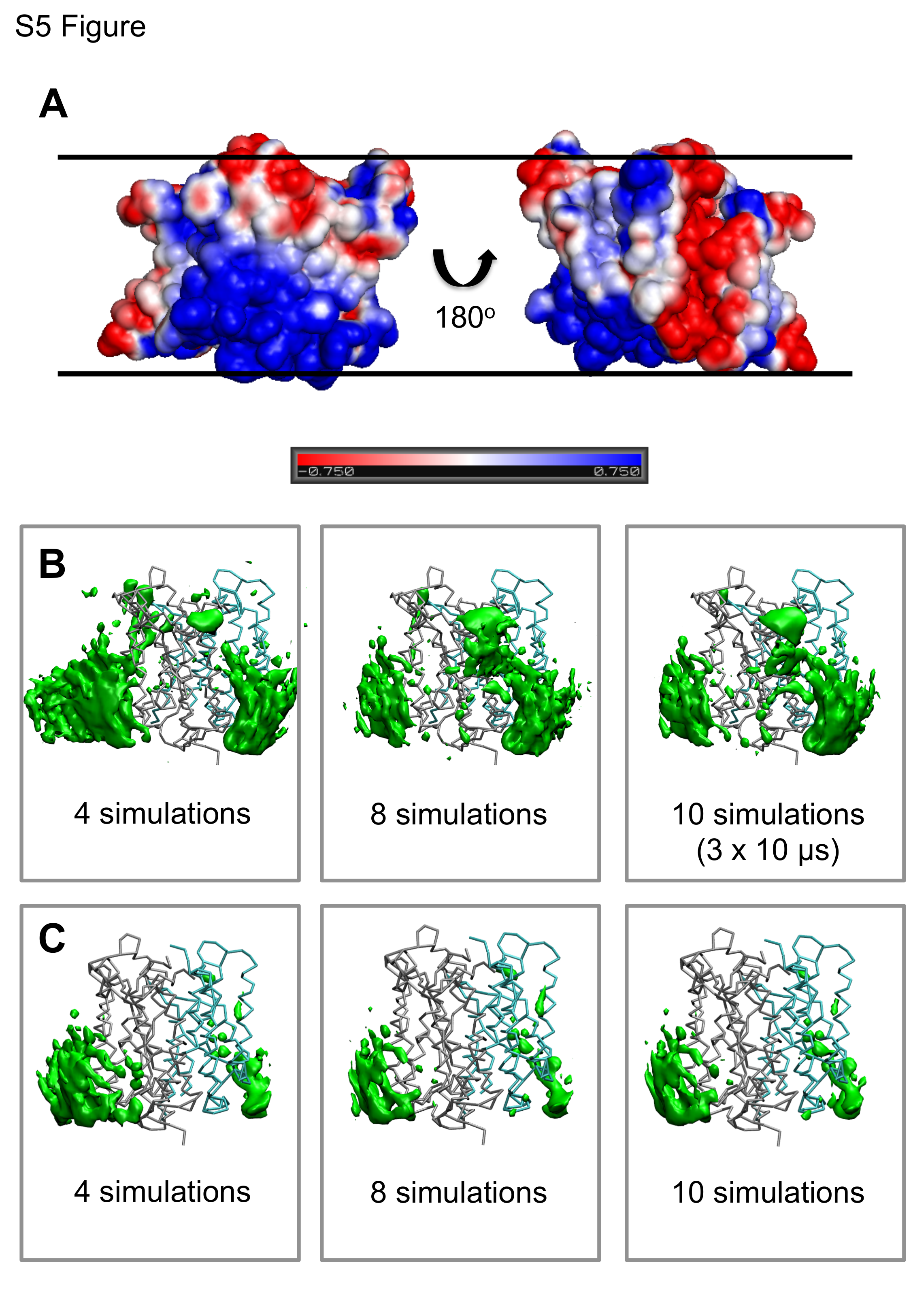

Supplement: S5 Fig — The black lines indicate the bilayer phosphate atoms. Blue indicates a positive surface and red a negative surface. The electrostatic calculation was performed using APBS [78] in PyMol [79]. Note that the basic cluster on cytosolic side of UraA is consistent with the “inside-positive” rule [80]. B,C. Convergence analysis. Occupancy of the CL molecules (green) in the simulations with the WT and a mutated form of the protein is shown by using 4, 8 and 10 repeat simulations. The systems used were chosen randomly. This analysis suggests that the simulation system converges after 4 or 5 repeat simulations. (TIF) [file pcbi.1004123.s005.tif]

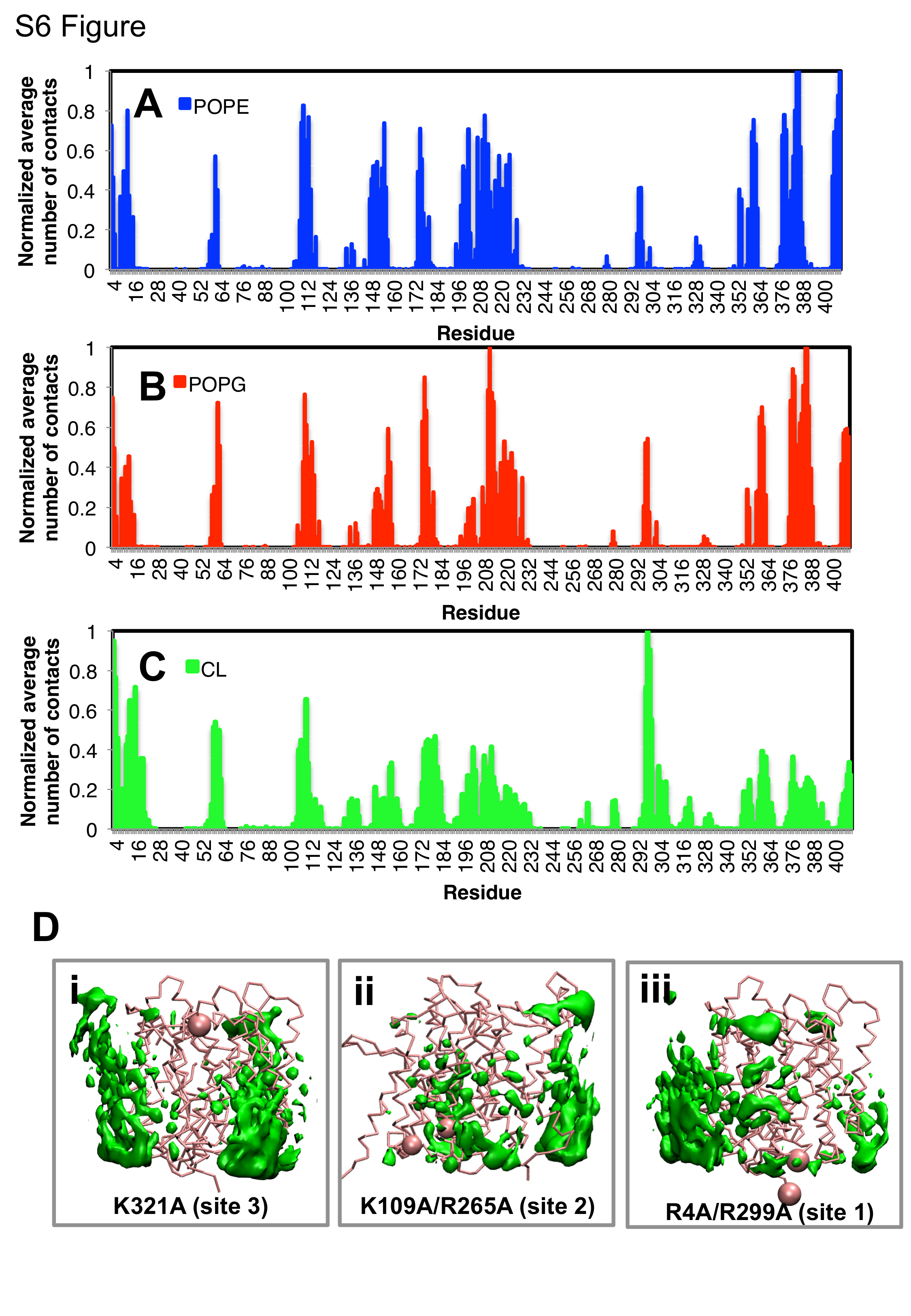

Supplement: S6 Fig — A,B,C. Normalized average number of contacts for the CG-MD simulations with the WT UraA. A cut-off distance of 7 Å for POPE and POPG and 8 Å for the CLs due to their largest headgroup was used. For the normalization, the number of contacts of a residue with a lipid type was divided by the largest number of contacts that the same lipid type made with any residue in the protein. This means that the residue with the most frequent contacts with a lipid type will have the value of 1 and the residue with the lowest contacts (or with no contacts) with a lipid type will have the value of 0. D. Occupancy plots showing the relative probability of occurrence of cardiolipin (CL; green), around UraA in the simulations with the following in silico mutations: (i) K321A, (ii) K109A/R265A, and (iii) R4A/R299A. The Cα atom of the mutated residues in each case is shown in van der Waals format. (TIF) [file pcbi.1004123.s006.tif]

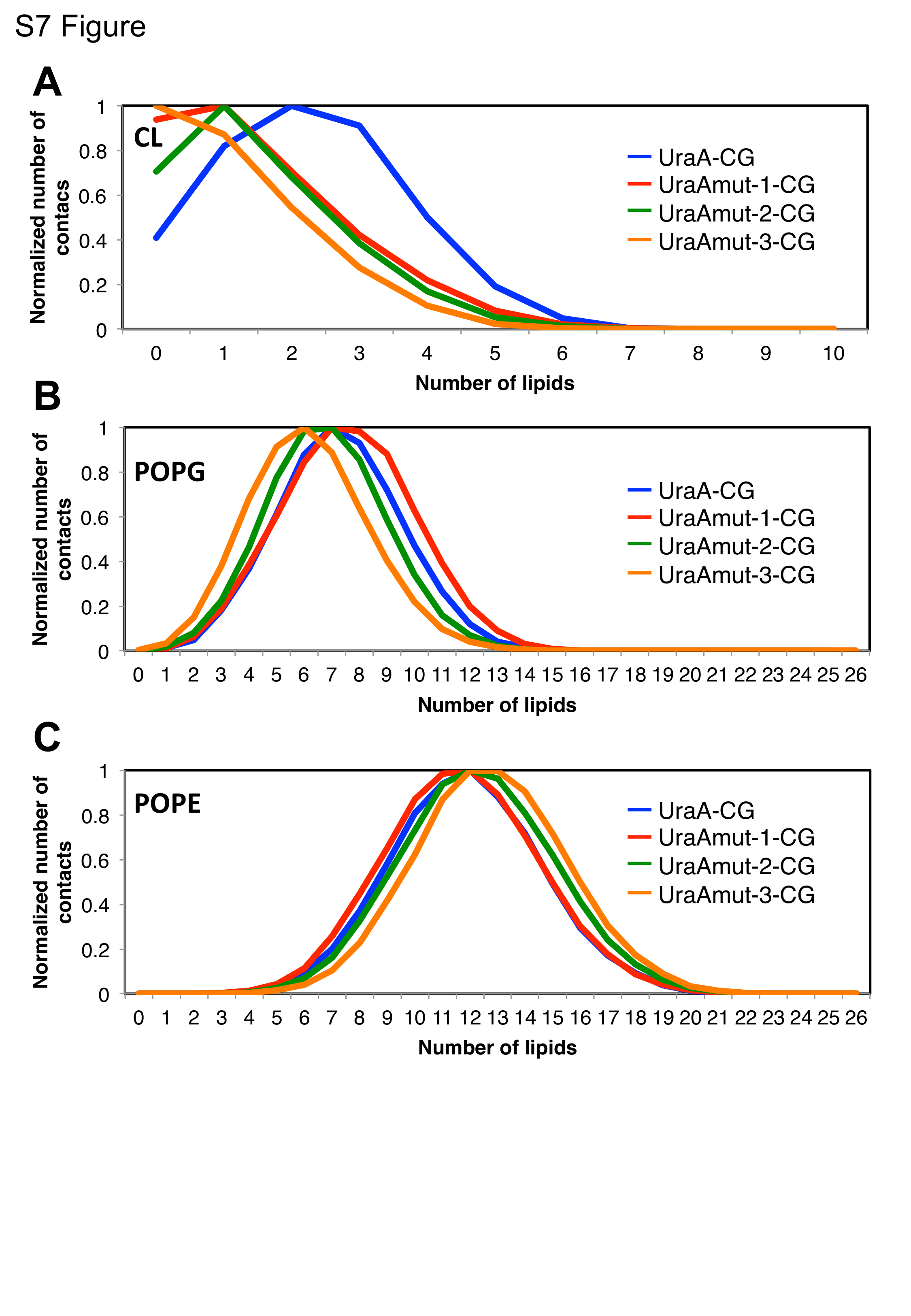

Supplement: S7 Fig — Number of CL (A), POPG (B) and POPE (C) lipids in contact with the UraA over the course of the simulations with the wild type and the mutated forms of UraA. The four different colors represent the four different simulation systems (UraA-CG in blue, UraAmut1-CG in red, UraAmut2-CG in green, UraAmut3-CG in orange; see Table 1). On average, UraA associates with ∼2 or 3 CLs, ∼8 POPGs and ∼12 POPEs in the simulations with the wild type protein. The number of CLs associated with the protein reduced to 1 or 2 in the simulations with the mutated forms (TIF) [file pcbi.1004123.s007.tif]

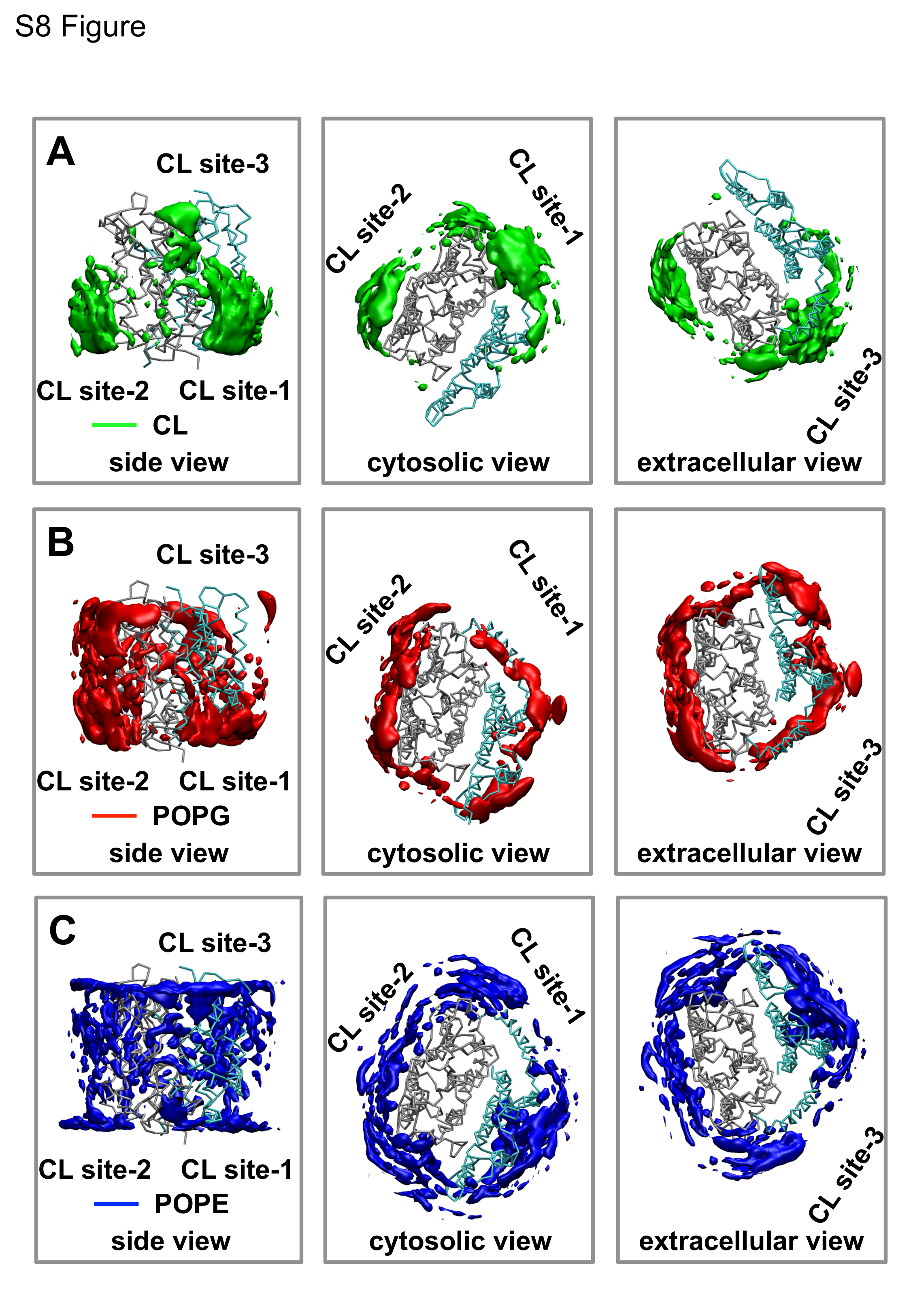

Supplement: S8 Fig — Occupancy plots showing the relative probability of occurrence of cardiolipin (CL; green), of the POPG (red) and of the POPE (blue) lipids around UraA. The occupancy was calculated as the average over all repeat coarse-grained simulations of the wild type UraA (UraA-CG in Table 1). Three different views are shown: a side view (left) showing the three CL binding sites, a cytosolic view facing the inner leaflet (middle) showing CL sites 1 and 2, and an extracellular view facing the outer leaflet of the bilayer (right) showing CL site 3. (TIF) [file pcbi.1004123.s008.tif]

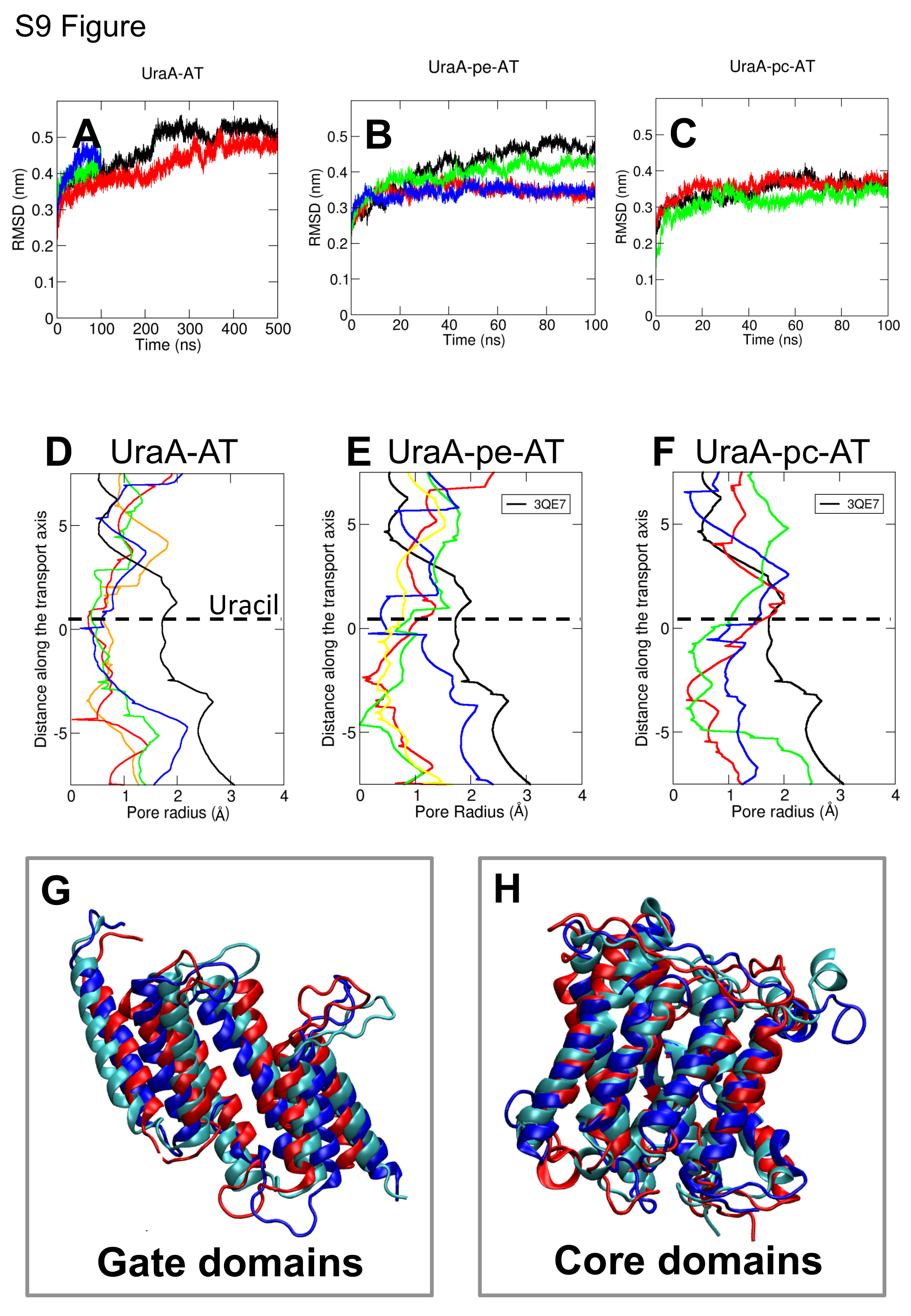

Supplement: S9 Fig — A,B,C. Root mean square deviation (RMSD) of UraA as a function of the simulation time for the atomistic systems (UraA-AT in PE, PG, CL, UraA-pc-AT in PC and UraA-pe-AT in PE in Table 1 and S1 Table). The RMSDs for the repeat simulations for each system are shown in different colors. D,E,F. Pore lining surfaces along the transport axis of the structures at the end of all the atomistic simulations (UraA-AT in D, UraA-pe-AT in E and UraA-pc-AT in F). The dotted line shows the uracil binding site as shown by Lu et al. [20] crystal structure. The profile was calculated using the program HOLE. The profile for each of the repeat simulations is shown in different colors. In all cases in the region between 0 to -5 nm along the transport axis the pore is closed (cytosolic side of the protein). After that region there is some variation in the profiles due to the present of more dynamic regions of the protein (e.g. dynamic loops of UraA and the end of the TM helices). The pore lining surface of the crystal structure is shown in black. G,H. Alignment of the gate (G) and core (H) domains from the end of the two extended simulations of the UraA-AT system with the corresponding domain in the crystal structure of UraA. Each simulation is shown in different color. The gate and the core domain from the crystal structure are shown in cyan. (TIF) [file pcbi.1004123.s009.tif]

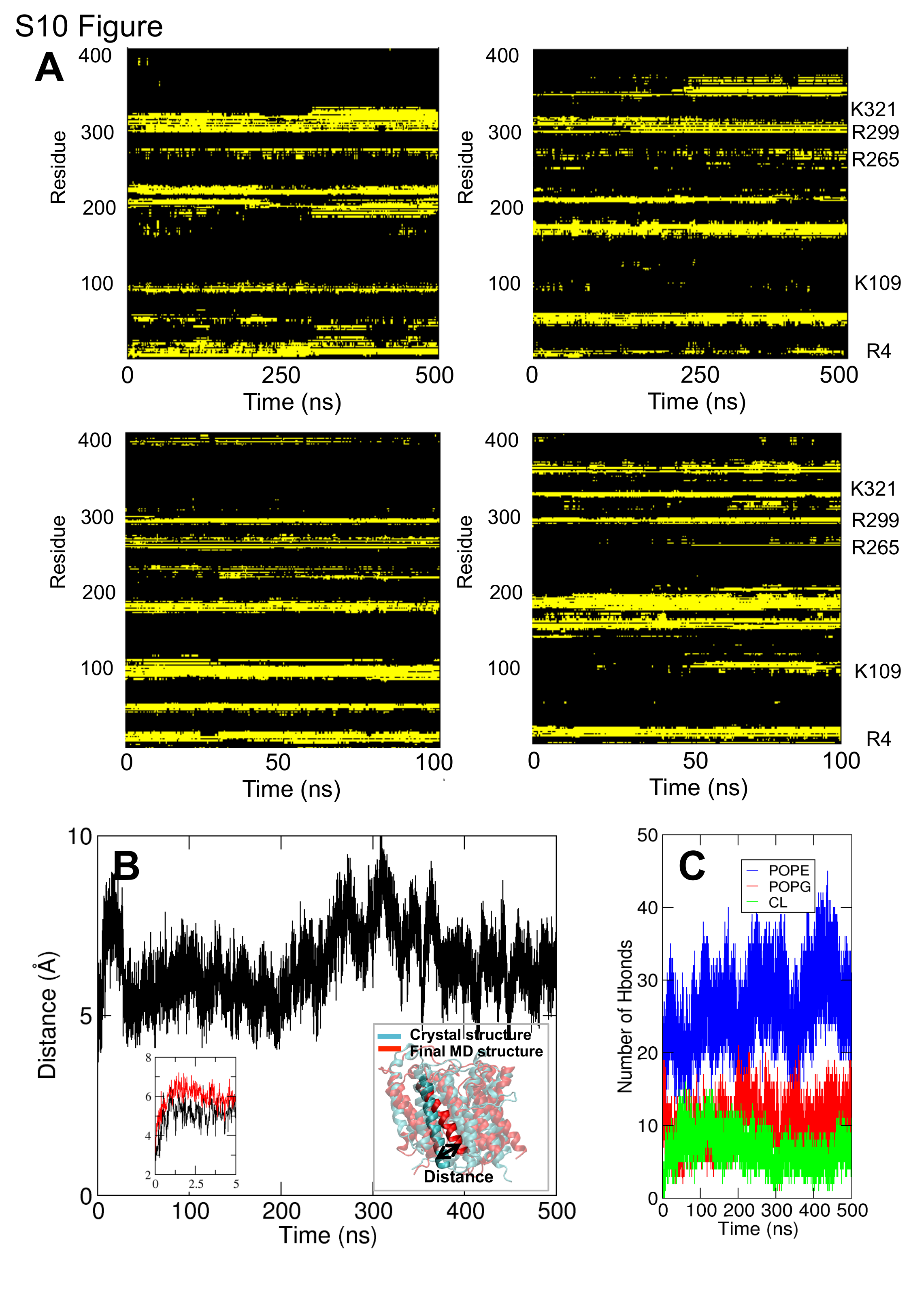

Supplement: S10 Fig — A. Interactions of UraA with CLs as a function of the simulation time for all the atomistic simulations with the wild type UraA (UraA-AT in Table 1). Yellow is used when the lipids are in contact with the protein and black when the lipids are not in contact. CL exhibit a prolonged interaction with specific residues in UraA (yellow). The positively charged residues identified in the CG-MD simulations to coordinate the CL binding to the three CL binding sites are also shown (see Fig. 3 in the main text). B. Distance between the residue 350 of the UraA crystal and the same residue of UraA in the atomistic simulations relative to time. To calculate the timescale of the changes in the position of the gate domain during the AT-MD simulation we have fitted the core domain of UraA in the extended atomistic simulations to the core domain of UraA crystal structure. Subsequently we have calculated the distance of residue 350 (Cα atom) of the crystal structure to the same residue in the simulation snapshots for all simulation frames. This residue was selected because is located on the cytosolic part of helix 12 (at the end of the helical region and close to the transportation path) and its movement would give a representative timescale for the movement of the cytosolic part of the gate domain relative to the crystal structure. This distance is shown schematically in the inset picture at the bottom right of the plot. The other inset picture shows the aforementioned distance for the first 5 ns of the two extended atomistic simulations. Note that the distance at the beginning of the atomistic simulations is not 0 because an initial movement of the gate domain relative to the core domain was observed during the CG-MD simulations. C. Number of H-bonds between the different lipids and UraA for one of the extended AT-MD simulations. (TIF) [file pcbi.1004123.s010.tif]

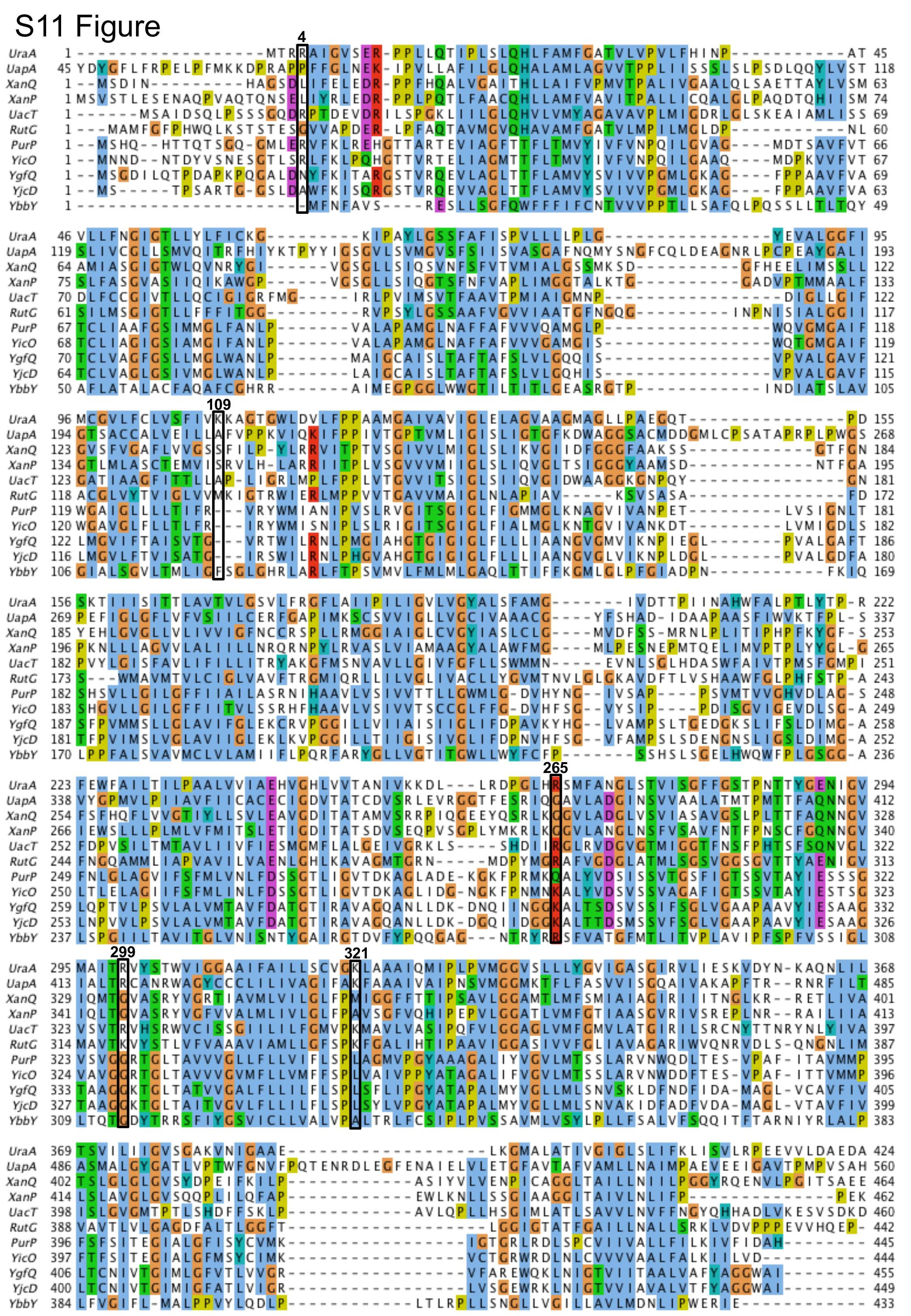

Supplement: S11 Fig — A. The basic residues on the CL binding sites are highlighted as boxes. The alignment was made using Clustal program in Jalview. The sequences are colored based on the sequence conservation using the ClystalX color scheme. (TIF) [file pcbi.1004123.s011.tif]
