# Supplementary material for: Molecular Dynamics Simulations of the Bacterial UraA H+-Uracil Symporter in Lipid Bilayers Reveal a Closed State and a Selective Interaction with Cardiolipin
Source: PLoS Comput Biol. 2015 Mar 2;11(3):e1004123. doi: 10.1371/journal.pcbi.1004123 (PMC4346270; doi:10.1371/journal.pcbi.1004123)
Supplement: S1 Table — (DOCX) [file pcbi.1004123.s012.docx]

**S1 Table: Summary of All Other Simulations**

| Simulation | Description | Bilayer | Duration |  |
| --- | --- | --- | --- | --- |
| UraA-pc-CG | UraA (PDB: 3QE7) | POPC (100%) | 1 x 1 µs |  |
| UraA-pe-CG | UraA (PDB: 3QE7) | POPE (100%) | 1 x 1 µs |  |
|  |  |  |  |  |
| UraAmut-4-CG | UraA [K321A] | POPE(75%)/POPG(20%)/CL(5%) | 10 x 1 µs | |
| UraAmut-5-CG | UraA [K109A, R265A] | POPE(75%)/POPG(20%)/CL(5%) | 10 x 1 µs | |
| UraAmut-6-CG | UraA [R4A, R299A] | POPE(75%)/POPG(20%)/CL(5%) | 10 x 1 µs | |
|  |  |  |  |  |
| UraA-pc-AT | UraA | POPC (100%) | 3 x 0.1 µs |  |
| UraA-pe-AT | UraA | POPE (100%) | 4 x 0.1 µs |  |
|  |  |  |  |  |
| pepgcl-CG | No protein | POPE(75%), POPG (20%), CL (5%) | 2 x 1 µs, 2 x 10 µs |  |
