# Supplementary material for: Molecular Dynamics Simulations of the Bacterial UraA H+-Uracil Symporter in Lipid Bilayers Reveal a Closed State and a Selective Interaction with Cardiolipin
Source: PLoS Comput Biol. 2015 Mar 2;11(3):e1004123. doi: 10.1371/journal.pcbi.1004123 (PMC4346270; doi:10.1371/journal.pcbi.1004123)
Supplement: S2 Table — (DOCX) [file pcbi.1004123.s013.docx]

**S2 Table: Lifetime of the interactions between the lipids and the 3 CL binding sites**

| Site | CL (ns) | POPG (ns) | POPE (ns) |
| --- | --- | --- | --- |
| 1 | sim1 = 241 (1)  sim2 = 634 (1)  sim3 = 647 (1) | sim1 = 156 (4)  sim2 = 255 (6)  sim3 = 179 (2) | sim1 = 77 (4)  sim2 = 168 (5)  sim3 = 240 (7) |
| 2 | sim1 = 234 (1)  sim2 = 155 (3)  sim3 = 497 (1) | sim1 = 80 (3)  sim2 = 74 (6)  sim3 = 120 (1) | sim1 = 63 (3)  sim2 = 76 (5)  sim 3 = 61 (3) |
| 3 | sim1 = 125 (1)  sim2 = 312 (1)  sim3 = 198 (1) | sim1 = 67 (3)  sim2 = 97 (2)  sim3 = 77 (3) | sim1 = 44 (6)  sim2 = 61 (4)  sim3 = 64 (7) |

Note that on S2 Table we show only the longest interaction time for which a binding site is continuously occupied by a lipid type for the 3 extended simulations (10 μs; sim1, sim2 and sim3) of the UraA-CG system. In parentheses we calculated the total number of changes of single lipids of the same lipid type in the CL binding site during the aforementioned time. See S2 Figure and S3 Figure for more information on the lifetime of interactions and Methods on how this analysis was performed.
